# Conserved Composition of Nod Factors and Exopolysaccharides Produced by Different Phylogenetic Lineage *Sinorhizobium* Strains Nodulating Soybean

**DOI:** 10.3389/fmicb.2018.02852

**Published:** 2018-11-26

**Authors:** Dan Wang, François Couderc, Chang Fu Tian, Wenjie Gu, Li Xue Liu, Verena Poinsot

**Affiliations:** ^1^Institute of Agricultural Resources and Environment, Guangdong Academy of Agricultural Sciences, Key Laboratory of Plant Nutrition and Fertilizer in South Region, Ministry of Agriculture, Guangdong Key Laboratory of Nutrient Cycling and Farmland Conservation, Guangzhou, China; ^2^Laboratoire des IMRCP, UMR5623 Université Paul Sabatier, CNRS, Toulouse, France; ^3^State Key Laboratory of Agrobiotechnology, and College of Biological Sciences, China Agricultural University, Beijing, China

**Keywords:** exopolysaccharide, mass spectrometry, Nod factors, *Sinorhizobium*, soybean

## Abstract

The structural variation of symbiotic signals released by rhizobia determines the specificity of their interaction with legume plants. Previous studies showed that *Sinorhizobium* strains from different phylogenetic lineages had different symbiotic performance on certain cultivated soybeans. Whether they released similar or different symbiotic signals remained unclear. In this study, we compared their *nod* and *exo* gene clusters and made a detailed structural analysis of Nod factors and EPS by ESI-MS/MS and two dimensions NMR. Even if there are some differences among *nod* or *exo* gene clusters; they produced much conserved Nod factor and EPS compositions. The Nod factors consist of a cocktail of β-(1, 4)-linked tri-, tetra-, and pentamers of N-acetyl-D-glucosamine (GlcNAc). The C2 position on the non-reducing terminal end is modified by a lipid chain that contains 16 or 18 atoms of carbon–with or without unsaturations-, and the C6 position on the reducing residue is decorated by a fucose or a 2-O-methylfucose. Their EPS are composed of glucose, galactose, glucuronic acid, pyruvic acid in the ratios 5:1:2:1 or 6:1:2:1. These findings indicate that soybean cultivar compatibility of *Sinorhizobium* strains does not result from Nod factor or EPS structure variations. The structure comparison of the soybean microbionts with other *Sinorhizobium* strains showed that Nod factor structures of soybean microbionts are much conserved, although there are no specific genes shared by the soybean microsymbionts. EPS produced by *Sinorhizobium* strains are different from those of *Bradyrhizobium*. All above is consistent with the previous deduction that Nod factor structures are related to host range, while those of EPS are connected with phylogeny.

## Introduction

Symbiotic nitrogen fixation is a critical process for the legume plant when the soil is poor in nitrogen. This occurs within the plant roots in a specialized organ: the nodule (Murray, 2011). The Rhizobium-legume interaction requires specific molecular signals between the symbiotic partners (Denarie et al., 1992; Cooper, 2007). When legume plants secret flavonoids into the soil, nodulation genes in rhizobia can be induced to synthesize the Nod factors (lipo-chitin oligosaccharides: LCOs) (Firmin et al., 1986). Such molecules have been identified for plenty of rhizobial species, as N-acetyl-D-Glucosamine (GlcNAc) oligosaccharides bearing diverse decorations (Perret et al., 2000). The structural variation of these metabolites determines the specificity of the interaction between microsymbionts and legume species (Lerouge et al., 1990; Roche et al., 1991; Sanjuan et al., 1992; Carlson et al., 1993; Lorquin et al., 1997a). Nod factors initiate the symbiotic program, while exopolysaccharides (EPS), lipopolysaccharides (LPS), capsular polysaccharides, and cyclic β-(1,2)-glucans play essential roles in the creation of the infection thread and in the nodule development (Fraysse et al., 2003; Cooper, 2007). Besides, a number of nodulation proteins secreted through type 3 secretion system (T3SS) could alter host signaling and suppress plant defenses. In several rhizobia, some of them were found to be involved in the specific nodulation of the host cultivars (Lopez-Baena et al., 2016).

Rhizobial exopolysaccharides (EPS) were reported as being heteropolysaccharides composed of common sugars almost bearing non-carbohydrate residues, such as pyruvyl, succinyl or acetyl groups (Gonzalez et al., 1996; Skorupska et al., 2006). The essential role of rhizobial EPS in establishing symbiosis was documented for the system forming indeterminate nodules (Parniske et al., 1994; Laus et al., 2004). However, the symbiotic function of EPS in determinate nodules is not the same case. Genetic and mutant phenotype analysis showed that EPS plays an important role in the symbiosis of *Bradyrhizobium japonicum*, but *S. fredii* HH103 EPS is not necessary for the effective nodule formation on soybean (Parniske et al., 1993, 1994; Rodriguez-Navarro et al., 2014). The EPS structures of fast growing and slow growing rhizobial strains are very different from each other. The subunit of the exopolysaccharides synthesized by strain *B. diazoefficiens* USDA110 is made up of D-glucose, D-mannose, D-galacturonic acid and D-galactose in a molar ratio of 2:1:1:1, bearing 4-O-methylation and acetylations (Janczarek, 2011). The structure of *S. fredii* HH103 EPS is composed of glucose, galactose and glucuronic acid, pyruvic acid in a molar ratio 5:2:2:1 and is partially acelated (Rodriguez-Navarro et al., 2014). Yet, information of EPS structure variation among closely related species cultured in the same condition has not been reported, to our knowledge. Nevertheless, these are crucial for understanding the functional evolution of complex carbohydrates.

Soybean is one of the most important legume crops in the world, and its symbiotic rhizobia belonging to *Bradyrhizobium* and *Sinorhizobium* genders have been widely isolated and investigated. Previous studies have shown that *Sinorhizobium* strains, SF45436 (*S. fredii* CCBAU45436), SF25509 (*S. fredii* CCBAU25509), SJ05684 (*S. sojae* CCBAU05684) and SS05631 (*S*. sp. III CCBAU05631), located in different phylogenetic lineages, showed different symbiotic compatibility with soybean cultivar. All of them were isolated from soybean nodules sampled from different sites in Chinese Huang-Huai-Hai Plain. Among these four strains, SF45436 can form nitrogen-fixing nodules on all tested cultivated soybeans, while other 3 strains showed uninfected pseudonodules with some commercial cultivars (Li et al., 2011a,b; Zhang et al., 2011, 2012; Tian et al., 2012; Zhao et al., 2018). Furthermore, biogeography analysis of soybean microbionts showed that *S. fredii* had a wider geographic distribution range than *S. sojae* and *S*. sp. III in the sampling location (Zhang et al., 2012). Comparative genomic analysis revealed that SJ05684 and SS05631 have smaller genomes than those of SF45436 and SF25509 (Tian et al., 2012). Zhao et al. inoculated the wild type strain of SF25509, SS05631, and SJ05684 on incompatible soybean cultivar JD17, and obtained some effective nodules except SJ05684. Analysis of the nodule isolates showed that those clones were mainly mutated in the coding region of Type 3 secretion system (Zhao et al., 2018). The results of genetic and mutants phenotype analysis showed that the nitrate-reduction gene cluster was absent in SJ05684 which is very essential for the symbiosis of SF45436 or SS05631 (Liu et al., 2017a). Here we were very interested in studying symbiotic signal structures of the four above strains. By analyzing and comparing gene clusters and symbiotic signal structures of four *Sinrohizobium* strains above (*S. fredii* CCBAU45436, *S. fredii* CCBAU25509, *S. sojae* CCBAU05684 and *S*. sp. III CCBAU05631), we aim to investigate whether the rhizobial symbiotic compatibility with soybean cultivar is determined by the variation present in their Nod factors or EPS structure. In the last part, structure comparison and gene phylogenetic analysis could offer the information on evolving mechanism of rhizobial symbiotic signals.

## Materials and methods

### Bacterial strains and media

The strains used in this study are *S. fredii* CCBAU45436, *S. fredii* CCBAU25509, *S. sojae* CCBAU05684 and *S. sp*. CCBAU05631. Rhizobium strains were grown at 30°C within either Tryptone Yeast extract (TY, with or without agar) (Beringer, 1974) or other media when it's mentioned. With Nod factor analysis, bacteria were developed in minimal liquid media (Vincent, 1970) supplemented with monosodium glutamate (1 g/L), monosodium succinate (2 g/L) and D-mannitol (3 g/L), here named VM media. This media has been also adopted to identify the EPS structure. For comparing EPS production of four rhizobia strains, Liquid Base medium has been also used: 10 g/L Tryptone, 5 g/L of Yeast extract, 2.5 mM CaCl_2_, 2.5 mM MgSO_4_ and 8.5 mM NaCl. All the liquid cultures have been incubated into a shaker at 120 rpm.

### Production and analysis of Nod factors

Three repeats of 3 L cultures of the four strains were done in parallel and the extraction and purification of Nod factors has been directly performed. Each single colony of bacteria on TY solid medium has been inoculated and grown with 5 mL of TY liquid medium for 24 h. 5 mL of cultured bacteria have been transferred afterwards into 100 mL of VM media (mentioned above) for 24 h more. 15 mL of subculture has been transferred to 1 L of VM media until OD_600_ reached 1.6–1.8 (about 20 h). Naringenin has been supplemented as nod gene inducer into culture at the concentration of 1.5 μM when OD_600_ reached about 0.2. Bacterial culture has been first centrifuged to remove the bacterial cells and then supernatant has been phase-partitioned against HPLC-grade 1-butanol. The volume ratio of supernatant and 1-butanol was 4:1. After stratification, 1-butanol layer has been transferred to an evaporation flask while the lower layer was phase-partitioned against 1-butanol for a second time. The twice collections of 1-butanol phase have been phase-partitioned against water at the volume ratio of 5:1. 1-butanol phase has been evaporated to dry and the residue suspended in water. The mixture has been then phase-partitioned against HPLC-grade Ethyl acetate at the volume ratio of 10:3. The water phase has been evaporated to dry and been re-dissolved by a small volume of 50% acetonitrile (ACN/H_2_O; v/v). The Nod factor crude extracts has been obtained in this way. The crude extracts have been concentrated to 600 μL of 50% acetonitrile (ACN/H_2_O; v/v) and the purification has been carried out by HPLC (Shimadzu LC-10AD Liquid Chromatography) using a C18 reversed-phase column (ODS2 equisorb, 25 cm × 7 mm, 5 μm, CIL-Cluzeau, France) with a flow rate of 2 mL/min. The UV detection occurred at 206 nm. The gradient was: 20% acetonitrile (ACN/H_2_O; v/v) for 15 min, the concentration has increased from 20 to 100% ACN in 20 min, finally 100% ACN for 5 min. The system has been conditioned again with 20% ACN for 7 min. The Nod factor elution range was 24–30 min. The fractions containing the Nod factor were evaporated and re-dissolved with 200 μL of 50% ACN and analyzed by ESI-MS.

### Production, extraction and purification of EPS

The supernatant containing the EPS has been centrifuged to eliminate cells and large cellular fragments. The high molecular weight (HMW) fraction of bacterial EPS has been precipitated by adding 3:1 volumes of 95% cold ethanol. Seven volumes more of ethanol have been added to the so obtained supernatant to allow the precipitation of the low molecular weight (LMW) fraction of EPS. The EPS fractions have been finally lyophilized.

### Glycosyl composition analyses

The EPS of each strain have been hydrolyzed in 2 M trifluoroacetic acid (TFA) at 110°C for 2 h. The TFA has been removed by repeated evaporation with isopropanol. The acidic monosaccharides have been methylated by diazomethane in methylether at room temperature. The derivation has been performed by silylation, with 20 μL pyridine, 100 μL TMCS-HMDS mixture (trimethylchlorosilane and 1,1,3,3,3-hexamethyldisilazane) at 70°C, for 1 h. Alternatively, the EPS have been methanolyzed with MeOH, HCl 3N, 90°C for 1 h. After evaporation, acetylation of the sugars occurred with 20 μL pyridine and 150 μL acetic anhydride at 70°C for 40 min. Derivatives of various monosaccharides as standards have also been prepared using the same protocols and analyzed by GC-MS in order to compare their retention times to the peaks obtained by analyzing the samples. Response factors of the different sugar types have been determined after derivatization of standards. These specific response coefficients have been then applied to quantify each monosaccharide family. GC-MS analyses of the EPS have been performed with 6890N GC interfaced with 5973 MSD by using a HP5-MS capillary column (25 m length 0.25 mm external diameter and 0.25 μm internal diameter). The oven program was 70 to 300°C in 57 min. The scanning masses have been in a range of m/z 50 amu to m/z 650 amu. The on-column injected volume was 0.1 μL. The source temperature was 230°C, the quadripole temperature set at 150°C, the He flow was 0.32 m/s.

### Structural analysis of EPS using nuclear magnetic resonance (NMR)

Nuclear magnetic resonance (^1^H NMR) spectroscopy has been performed on a Brücker AMX500 spectrometer (Wissenbourg, France). The samples have been dissolved in D_2_O (100%). NMR spectra have been recorded at 303 K, at 500 MHz (^1^H) and 125.75 MHz (^13^C) using a cryoprobe. Chemical shifts are given in ppm after automatic calibration on the solvent. ^1^H spectra have been recorded for all the EPS. Homonuclear experiments have been performed: COZY and selective NOE. Finally Heteronuclear ^1^H-^13^C HSQC and HMBC studies have been done on the most abundant and resolved sample. The spectra were layed out using MestResNova.

### ESI-ToF mass spectrometry

Fractions of *Sinorhizobium* strains LMW EPS have been analyzed in the negative-ion mode on a QqTOF system (Ultima, Waters). Probe: 3 kV, cone 50 V, Rf lens 25 V and collision: 10 V, up to 25 V for MS/MS. Samples have been dissolved at a concentration of 2 mg/mL in water/methanol 1:1 with 0.1% ammonia. The injection has been carried out with a flow rate of 7 μL/min. The scan range has been established between m/z 500 and m/z 2,500 in 2 s with 0.2 s interscan delay. The spectra have been deconvoluted using Max Ent 3.

### Phylogenetic analysis

Single gene alignments were performed with molecular evolutionary genetics analysis (MEGA) (Tamura et al., 2011). The neighbor-joining trees were constructed by using the same software, and 1,000 bootstraps were done.

## Results

### Genes involved in Nod factor biosynthesis

**Figure 1:**
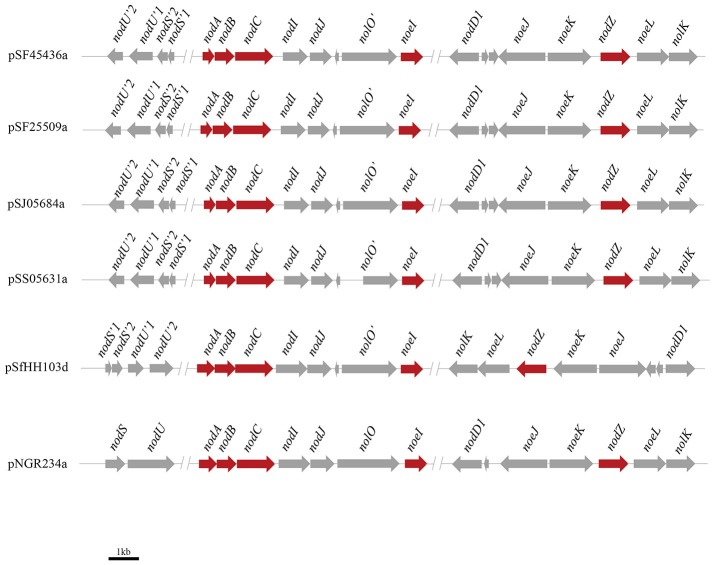
Genetic organization of *nod* gene clusters on Symbiotic plasmids in four *Sinrhizobium* strains. pSF45436a, pSF25509a, pSJ05684a, pSS05631a, pSfHH103d, and pNGR234a represent the symbiotic plasmid from *S. fredii* CCBAU 45436, *S. fredii* CCBAU25509, *S. sojae* CCBAU 05684, *S*. sp. CCBAU 05631, *S. fredii* HH103 and *S*. sp. NGR234, respectively.

In previous studies strains SF45436 (*S. fredii* CCBAU45436), SF25509 (*S. fredii* CCBAU25509), SJ05684 (*S. sojae* CCBAU05684) and SS05631 (*S*. sp. III CCBAU05631) were isolated from nodules of soybean distributed in Huang-Huai-Hai Plain, where *Sinorhizobium* strains were reported as being the dominant population of the soybean microbionts in such region (Li et al., 2011a,b; Zhang et al., 2011, 2012; Tian et al., 2012). Among these four strains, SF45436 can form nitrogen-fixing nodules on all tested soybean cultivar, while the other 3 strains showed uninfected pseudonodules with some commercial cultivar (Li et al., 2011a,b; Zhang et al., 2011, 2012; Tian et al., 2012; Liu et al., 2017a; Zhao et al., 2018). Their host range was summarized in the Table 1. The genome sequences of SF45436, SF25509, SJ05684, and SS05631 have been completed. The genome sizes of SJ05684 (6.06 Mb) and SS05631 (6.35 Mb) are smaller than those of SF45436 (6.87 Mb) and SF25509 (6.77 Mb) (Tian et al., 2012). To investigate whether the strains having different soybean cultivar host range release different symbiotic signals, the genes involved in synthesis of Nod factors were first extensively studied. As shown in Figure 1, the proposed nodulation gene clusters present on symbiotic plasmid A of the four strains are identical, including the genes encoding for the chitinic core (*nodABC)*, the fucosylation (*nodZ*), methylfucosylation (*noeI*), the Nod factor exporters (*nodIJ*), the mannose-6-phosphate isomerase (*noeJ)*, the phosphomanomutase (*noeK*), the GDP-mannose 4,6-dehydratase (*noeL*) and the nucleotide sugar epimerase (*nolK*). Gene analysis revealed that there are two open reading frames (ORFs) of *nodU* homologous to that of *S*. sp. NGR234 in the four analyzed strains, designated as *nodU'1* and *nodU'2*, respectively. Similarly, there are two ORFs of *nodS* homologous to that of strain NGR234 as well (Freiberg et al., 1997). However, DNA alignments showed that *nodS'1* (from SF45509, SF25509, SJ05684, SS05631, and HH103), *nodU'1* (from SF45509, SF25509, SJ05684, SS05631, and HH103) and *nolO* homologous nucleotides (from SF45509, SF25509, SJ05684, SS05631, and HH103) have −1 frame shift mutation, indicating these strains have truncated versions of *nodSU* (encoding the N-methylation and the carbamoylation, respectively) and *nolO* (predicted carbamoyl transferase) genes (Figure 1, Figures S1–S3) (Vinardell et al., 2015). As usually, the Nod factor regulatory gene *nodD1* is also located on the same symbiotic plasmid. Furthermore, the *nodE* gene of the four strains are all located on the chromosome. Yet, SJ05684 doesn't have *nodP* or *nodQ* genes on symbiotic plasmid B. These two genes are reported to encode the sulfation of lipo-oligosaccharide signals (Schwedock and Long, 1990; Roche et al., 1991).

**Table 1:** Summary of host range by four *Sinorhizobium* strains (CCBAU45436, CCBAU25509, CCBAU05684, CCBAU05631).

	**Soybean cultivar (*****Glycine max*****)**				
**Strains**	**NN86-4**	**JD17**	**KF16**	**C08**	**LQ**
SF45436	+	+	+	+	+
SF25509	–	–	+	–	+
SJ05684	–	–	–	–	+
SS05631	–	–	+	–	+

*SF45436 indicates S. fredii CCBAU45436; SF25509 indicates S. fredii CCBAU25509; SJ05684 indicates S. sojae CCBAU05684; SS05631 indicates S. sp. CCBAU05631*.

*+ indicates normal nodule; – indicates uninfected nodule*.

### Nod factor structure analysis

**Figure 2:**
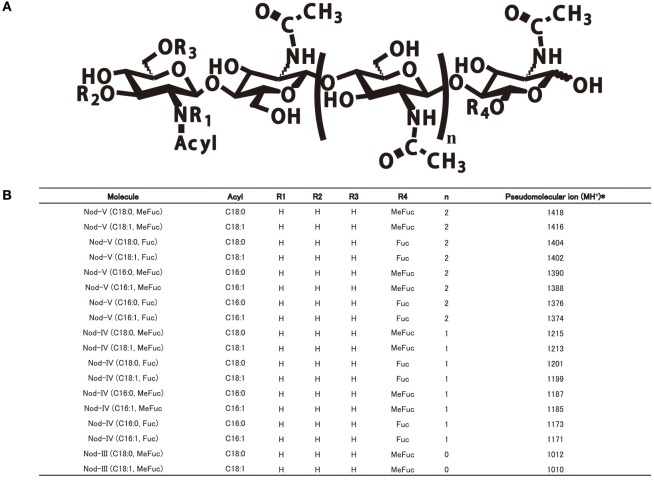
The common Nod factor structures with diverse residues of four *Sinorhizobium* strains on the reducing or non-reducing ends. **(A)** The common Nod factor structures with diverse residues on the reducing or non-reducing ends. **(B)** Summary of ESI-MS/MS analysis of LCOs secreted by four *Sinorhizobium* strains (CCBAU45436, CCBAU25509, CCBAU05684, CCBAU05631).

In order to determine the Nod factor structure of the four sinorhizobia, we cultured the bacteria into VM media supplemented with naringenin as *nod* genes inducer and extracted the LCOs by 1-butanol. After HPLC purification, the chemical structures and compositions of the LCOs were analyzed with help of positive mode ESI-MS/MS and ESI-TOF MS (Figures S4A–D). The Nod factors secreted by these four strains showed the same compositions, according to MS and MS/MS analyzes. These consist in a cocktail of β-(1, 4)-linked N-acetyl-D-glucosamine (GlcNAc) tetramers and pentamers. The C2 position on the non-reducing terminal end is modified by a lipid chain contains 16 or 18 atoms of carbon with or without unsaturated bonds (C16:1, C16:0, C18:1, C18:0). On the reducing end of the chitin backbone, the C6 position is decorated by a fucose or a methyl-fucose (Figures 2A,B). To assess that the deoxysugar observed on the MS/MS spectrum is a fucose and not a rhamnose, we made the glycosyl analysis of the isolated the Nod factors. For this purpose, we hydrolyzed the compounds with methanolic HCl 3N and performed an acetylation of the so released monosaccharides. The GC/MS analysis (spectrum and retention time) clearly demonstrated the fucose nature of this residue (Data not shown). Location of the methyl on the fucose was also reported to be on O-2 in other LCOs produced by *S. fredii* (Bec Ferte et al., 1996; Gil-Serrano et al., 1997). The tetramers are the major compounds secreted by all of the four strains, the pentamers and trimers are present in significant amounts, but less abundant. The LCOs structures of four strains are very similar to those of *S. fredii* HH103 and *S. fredii* USDA257. These are not sulfated, contrarily to what has been reported for the Nod factors of *Sinorhizobium* sp. NGR234 (Price et al., 1992; Bec Ferte et al., 1996; Gil-Serrano et al., 1997; Pueppke and Broughton, 1999).

### Genes involved in exopolysaccharide biosynthesis

**Figure 3:**
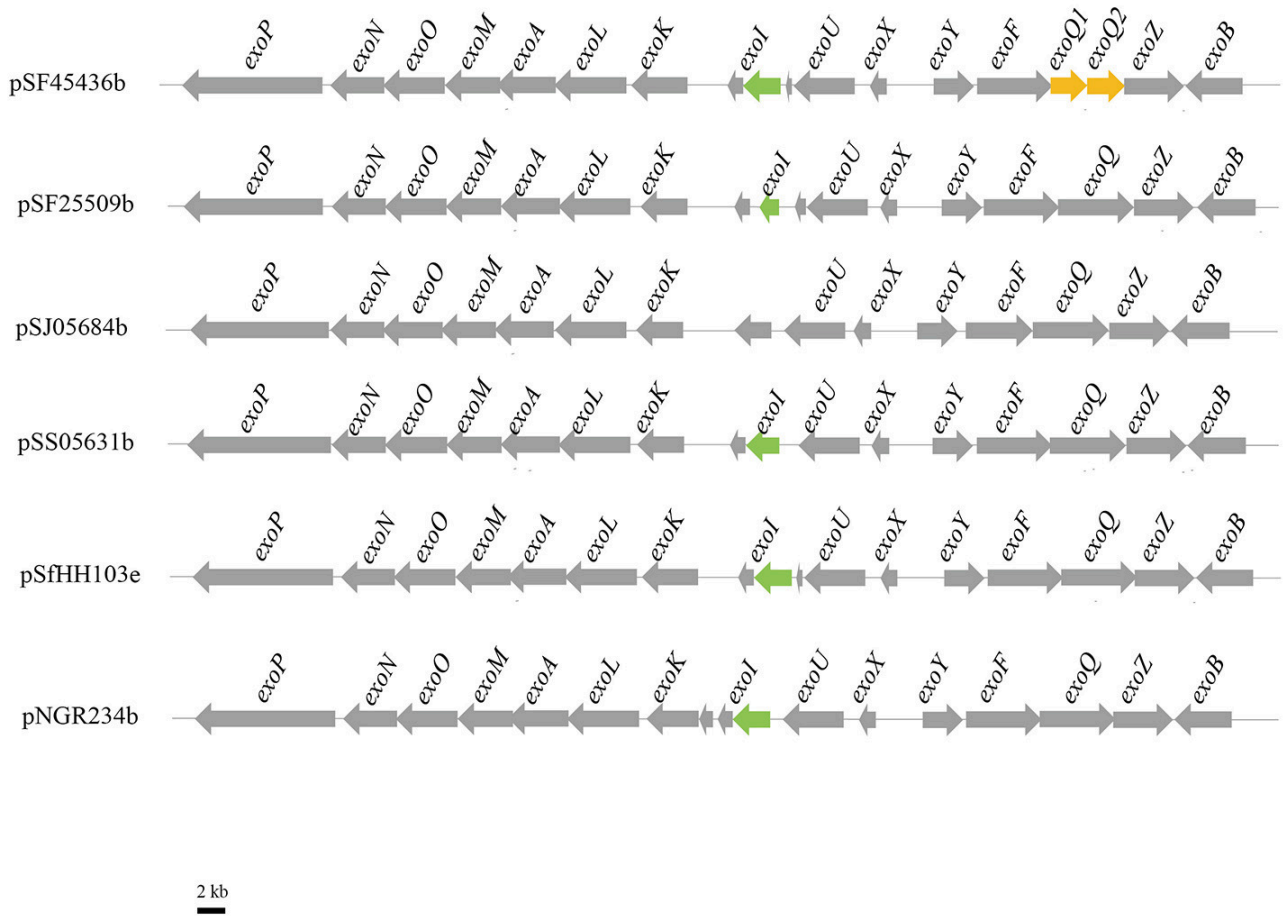
Genetic organization of *exo* region on Symbiotic plasmids in four *Sinrhizobium* strains. pSF45436b, pSF25509b, pSJ05684b, pSS05631b, pSfHH103d, and pNGR234a represent the symbiotic plasmid from *S. fredii* CCBAU45436, S. *fredii* CCBAU25509, *S. sojae* CCBAU05684, *S*. sp. CCBAU05631, *S. fredii* HH103, and *S*. sp. NGR234, respectively.

Exopolysaccharides (EPS) are reported to protect the bacteria against the host defense system during the plant-microbe interaction. EPS structural variations among closely related species having different host cultivar compatibility are not well-documented. Therefore, we would like here to analyze the EPS structures of the four strains. Most genes involved in EPS biosynthesis of the here tested *Sinorzhiobium* strains are located on the large symbiotic plasmid B. The *exo* clusters of four strains mainly contain *exoPNOMALKIUXYFQZB*. It is interesting to note that SJ05684 lacks the *exoI* gene (Figure 3). *exoI* encodes for succinoglycan biosynthesis protein, but its mechanism remains unknown. There are two open reading frames of *exoQ* in SF45436, even if the others have only one. It was reported *exoA, exoL, exoM, exoO*, and *exoU* are glucosyl transferases while *exoF, exoP, exoQ*, and *exoK* are responsible for EPS polymerization and transportation. *exoB, exoN, exoY*, and *exoZ* encode the UDP-glucose-4-epimerase, UDP glucose synthase, galactosyltransferase and acetyltransferase, respectively. As shown in Figure 3, the *exo* clusters of four strains are much similar to those in *S. fredii* HH103 or *S*. sp. NGR234 (Schmeisser et al., 2009; Rodriguez-Navarro et al., 2014). For the EPS synthesis regulators, *exoR, exoS, exoD*, and *expR* of *S. meliloti* homologous genes are located on the chromosomes of all four strains. The genes *exsB* and *wggR* are on the symbiotic plasmids. Two copies of *mucR* genes are found on both chromosomes and symbiotic plasmids of the four strains.

### Glycosyl composition analyses of EPS

To compare the EPS sugar compositions, we first cultured the strains in Liquid Base medium containing abundant sources of carbon and nitrogen (Gharzouli et al., 2013). After precipitation with different proportions of ethanol, high molecular weight and low molecular weight fractions of EPS were obtained and the quantities issued from different strains are summarized in Table 2. Using such LB medium, the biggest quantity of total EPS was produced by SS05631 and the lowest by SF45436.

**Table 2:** Production of EPS in four *Sinorhizobium* strains.

	Mean production of EPS (mg/OD_600_ of bacteria) ± SD^*^		
**Rhizobia strain**	**LMW EPS**	**HMW EPS**	**Total EPS**
SF45436	20.9 ± 4.8	45.7 ± 5.7	66.5 ± 9
SF25509	14.1 ± 3.9	59.7 ± 7.8	73.8 ± 8.7
SJ05684	33.9 ± 15.3	46.8 ± 5.3	80.7 ± 19.5
SS05631	44 ± 16.8	61.3 ± 2.6	105.4 ± 18

* *SD, standard deviations. LMW means low molecular exopolysaccharides (molecular masses less than 10 kDa); HMW means high molecular weight exopolysaccharides (molecular masses over 10 kDa). The quantity of liquid medium is 500 ml per culture. SF45436 indicates S. fredii CCBAU45436; SF25509 indicates S. fredii CCBAU25509; SJ05684 indicates S. sojae CCBAU05684; SS05631 indicates S. sp. CCBAU05631*.

The sugar identification was first carried out using EI^+^ GC/MS, by comparing the retention times and mass spectra to standards. The EPS of four strains are made up mainly of glucose (Glc), galactose (Gal), glucuronic acid (GlcA), and mannose (Man) (Table 3). The silylation strongly underestimated the uronic acid content but allowed a good separation of the hexoses. Methanolysis and acetylation gave access to the uronic acid nature and ratio. In the mean, a proportion of 6–7 Glc for 1 Gal and 2GlcA was found. The LMW EPS of two strains (SF45436 and SJ05684) were submitted to complete NMR an ESI-MS/MS studies that confirmed these results. Even if the purification has been performed through two-step precipitation, the produced fractions appeared to be reasonably clean. Actually, the different hydrolyses (TFA, Acetic acid, HCl-methanolysis) followed by GC/MS analyzes revealed neither amino acids nor ribose or lipids. Moreover, we made proteinase K digestions of the collected EPS without loss of weight. ESI-MS analyzes have been performed in the negative and positive ionization modes. These exhibited only saccharides in the m/z 700–2,500 domain. The combination of bad ionization potentials and low solubility of bigger carbohydrates could explain the absence in our ions in the higher mass ranges.

**Table 3:** Monosaccharide composition of *Sinorhizobium* strains EPS cultured in rich media as determined by GC-MS.

	**Monosaccharide compostion (%)**^**a**^		
**EPS and strain**	**Glc (t_R_ = 35.9; 22.0)**	**Gal (t_R_ = 33.1; 19.5)**	**Man^a^ (t_R_ = 30.1; 18.6)**	**GlcA^b^ (t_R_ = 36.8; 20.8)**
**LMW EPS**				
SF45436	69.3 ± 0.3	9.7 ± 0.3	1.0 ± 0.1	20.0 ± 0.9
SF25509	65.5 ± 5.3	10.9 ± 2.3	2.2 ± 3.0	21.4 ± 4.5
SJ05684	64.5 ± 0.6	10.7 ± 0.4	4.3 ± 1.1	20.5 ± 0.7
SS05631	68.9 ± 0.1	7.9 ± 0.3	1.0 ± 0.1	22.2 ± 0.2
**HMW EPS**				
SF45436	45.5 ± 1.5	22.7 ± 1.2	20.4 ± 2.8	11.4 ± 1.9
SF25509	55.7 ± 0.2	16.3 ± 0.2	11.0 ± 0.1	17.0 ± 0.4
SJ05684	20.4 ± 0.4	22.5 ± 1.5	45.6 ± 2	11.5 ± 2.2
SS05631	44.5 ± 0.2	21.7 ± 0.1	23.0 ± 0.2	10.8 ± 0.5

*Glc, Glucose; Gal, Galactose; Man, Mannose; Gal A, Galacturonic Acid; t_R_, retention time of each monosaccharide on GC-MS (the first value is the TMS derivative most intense peak and the second to the methylated and acetylated one). Values are expressed as means pooling all the results ± standard deviations where applicable. SF45436 indicates S. fredii CCBAU45436; SF25509 indicates S. fredii CCBAU25509; SJ05684 indicates S. sojae CCBAU05684; SS05631 indicates S. sp. CCBAU05631*.

a *The proportion of mannose in LMW EPS represents it's from HMW EPS because of second-step- precipitation*.

b *Gal A demonstrates a bad response factor when derivatized with TMS, its value is obtained only after methylation and acetylation*.

### The four strains exhibit similar NMR profiles of EPS

**Figure 4:**
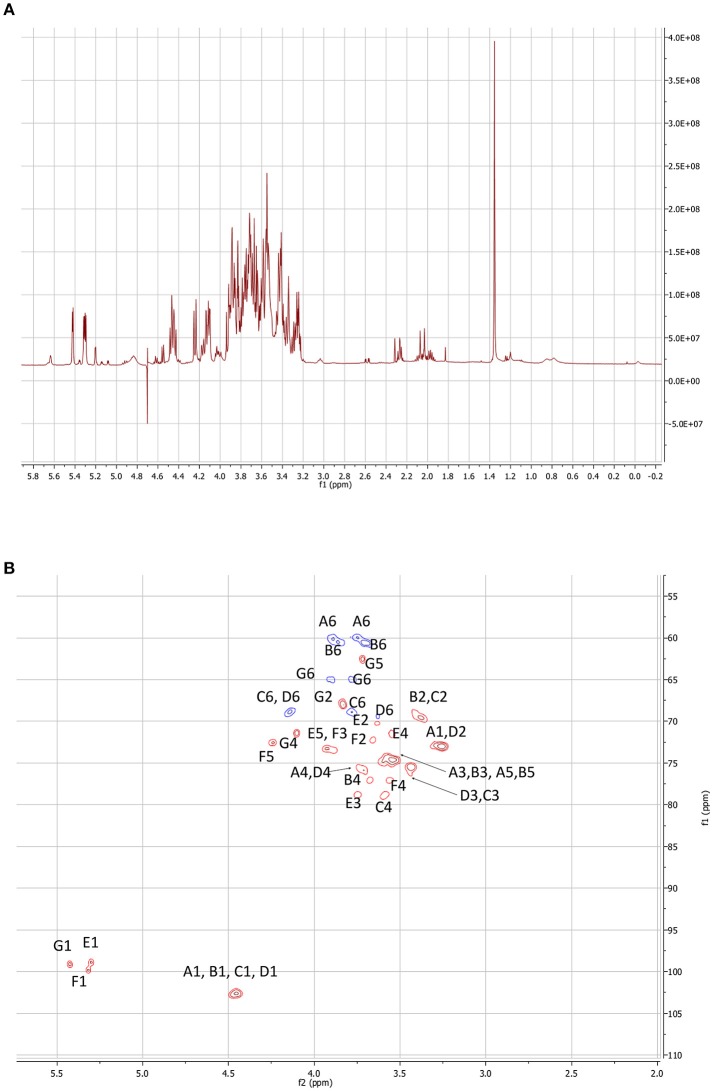
NMR experiments performed on the LMW EPSs of *S. fredii* CCBAU45436. **(A)**^1^H spectrum of the LMW fraction. **(B)** HSQC map of the carbohydrate signal domain. The letters correspond to a sugar unit and the number to the location on the sugar.

The ^1^H spectra of all the collected LMW EPS has been recorded and compared within the same species (two *S. fredii* strains) or among different ones (*S. fredii, S. sojae*, and *S*. sp.). As described later, the EPS ethanol precipitation allows obtaining a fraction mostly constituted of an octasaccharidic repeating subunit. The samples issued from different strains produced very similar ^1^H NMR spectra. In order to assess this affirmation, two samples (one belonging to *S. fredii* and the other to *S. sojae*) were submitted to complete NMR studies. The ^1^H NMR spectrum of the studied strain (Figure 4A) indicated that the samples are sufficiently clean and dissolved to allow their 2D analyses.

**Figure 5:**
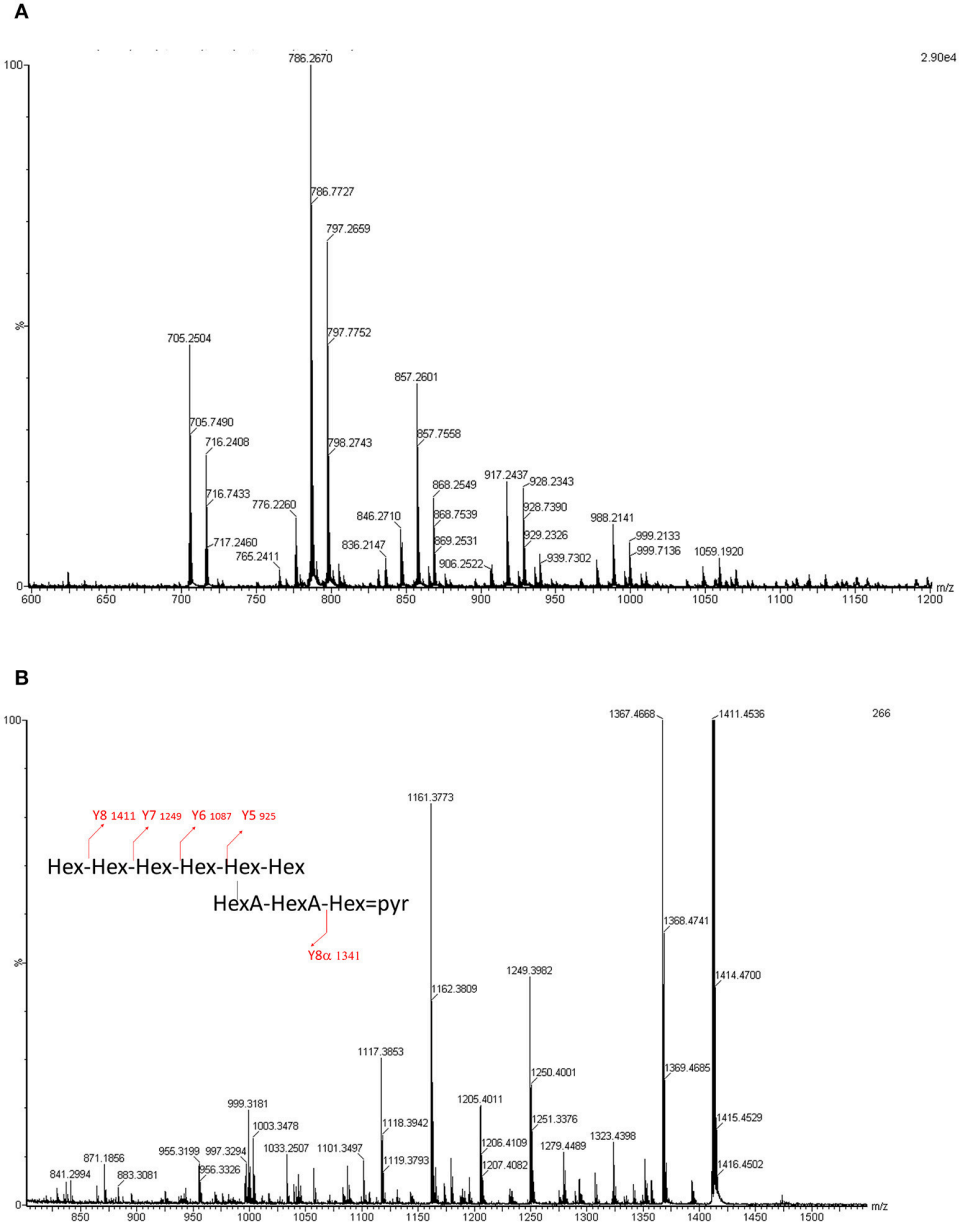
**(A)** ESI-MS profile of the LMW EPSs obtained for *Sinorzhibium* strains (CCBAU45436 and CCBAU05684). Acquisition domain ranges from 500 to 1,500 Da. Two species can be observed, one octa (m/z 705.27) and one non-asaccharide (m/z 786.27). The other peaks are sodium phosphate adducts. **(B)** MS/MS spectrum of the m/z 786 molecular ion. The fragments and the degradation pattern is given in Table S1.

The 2D maps were identical. We will detail the analysis of SF45436 EPS here. Considering the 1–2.5 ppm domain of the ^1^H spectrum, even if the spectrum is a bit crowded due to residual medium contaminants, no succinate (doublet at 2.05 ppm) and no acetate (singlet at 1.8 ppm) signals could be detected. This indicated that the EPS are only bearing pyruvate as confirmed by the MS spectra (Figure 5). Two Dimensional NMR analyses allow to determine the structure of carbohydrates and to assess most of the linkages in the polysaccharide. ^1^H-^1^H Homonuclear experiments are sensitive and will give access to neighbor-neighbor information. NOESY sequence is enabling spacial neighboring, when COZY sequence is enabling the determination of bounded proximity. Selective irradiation of isolated signals allowed us, using a NOE sequence, to find the sequence of the monosacharides. The “selfish” signals (at 5.42; 5.31; 4.84; 4.58; 4.46; 4.24 and 3.26 ppm) were irradiated and the signals in spatial interaction with each of them recorded. It is of interest to note that the signals at 4.84 and 4.58 ppm do not appear very well on Figure 4A due to the saturation of the water signal at 4.7 ppm.

Heteronuclear correlations enable to obtain two types of information. From the HSQC experiment, it is possible to attribute to each ^1^H signal the corresponding ^13^C chemical shift through a ^1^*J* correlation (Figure 4B). Moreover, this enables to establish the conformation of the sugars. Considering the anomeric domain (ranging from 4.3 to 5.5 ppm for ^1^H and from 97 to 105 ppm for ^13^C) it clearly appears that 3 of the hexoses are in alpha configuration where the others are all in beta configuration. Actually, alpha configuration are characterized by elevated ^1^H chemical shifts associated to low ^3^J_H1, H2_ constant (5–12 Hz). Only HMBC experiments are allowing establishing the sequence of the heteropolymer. Actually, mostly through ^3^*J* coupling, it gives access, starting with a given ^1^H signal, to its neighbored ^13^Cs (even through the glycosidic junction). Reversely, starting from a well-defined ^13^C signal it allows finding its ^1^H neighbors. COZY maps enabled to find out the order between the neighbored carbons by indicating the ^1^H bound to the carbon located in alpha.

The compilation of these different data gave us access to the complete structure. The ^1^H and ^13^C chemical shifts for each sugar subunit of the polysaccharide are given in Table S1.

Starting with the pyruvate signal, associated galactose ^1^H and ^13^C shifts could be found (sugar G). Actually, the ^13^C of the pyruvate signal at 100.1 ppm correlates with the H6 and H4 of the bounded sugar (on the HMBC map). These are correlated to the corresponding carbon (C6 and C4) using the HSQC map. Starting with these signals (^1^H and ^13^C), H5 and C5 are found and sometimes H3, C3 and so on. The anomeric signal of this sugar unit here at 5.43 ppm gave access to the supplementary bounded carbon belonging to the next connected sugar unit (3.56/77 ppm). This signal was found out to be the C4 of a glucuronic acid subunit. Again this acidic carbohydrate is sequenced by crossing COZY, HMBC, and HSQC data. The C1 of this uronic acid unit is connected to a signal at 3.79/79.0 ppm that can be attributed this time to the C3 of another GlcA unit.

The same approach has been used starting with the C6 signals of the hexoses (CH_2_ appearing in blue on the HSQC surface -where the CH are in red-). Considering their elevated chemical shifts (over 4 ppm for ^1^H and around 69 ppm for ^13^C) it appeared that two of them are involved in glycosidic junctions. The coupling constants and chemical shifts of the proton signals have shown that these Hexoses were all glucoses as the ^3^J_H4, H5_ are all around 10 Hz. Some were bounded in 1->6, the other in 1->4, this can easily be found out because the C6 presents chemical shifts around 60 ppm when free and close to 70 ppm when they are bounded.

Even if mass spectrometry clearly indicated the predominant presence of an octasaccharide, only seven different sugars could be identified using high resolution NMR. Therefore, as confirmed by the intensity of several ^1^H signals, one of them must be present identically within the repeated in the subunit.

### ESI-QqToF MS/MS analysis of S*. fredii* CCBAU45436 LMW EPS

Using ESI-QqToF MS experiments we determined nine doubly charged ions, which correspond to sodium or sodium phosphate adducts of the molecular ions, where only two ions at m/z 786.27 and m/z 705.24 corresponded to the molecular ions [M-2H]^2−^. These masses are consistent with respectively a nona- and an octa-saccharide carrying a pyruvyl group and composed of 6 or 7 hexoses and two hexuronic acids (Figure 5A).

The fragmentation of the [M-2H]^2−^ ion at m/z 786.27 has been analyzed using ESI- QqToF MS/MS experiments. The spectra has shown mainly doubly or singly charged ions with a Y fragmentation pattern. Some X ions have also been observed (Figure 5B).

**Figure 6:**
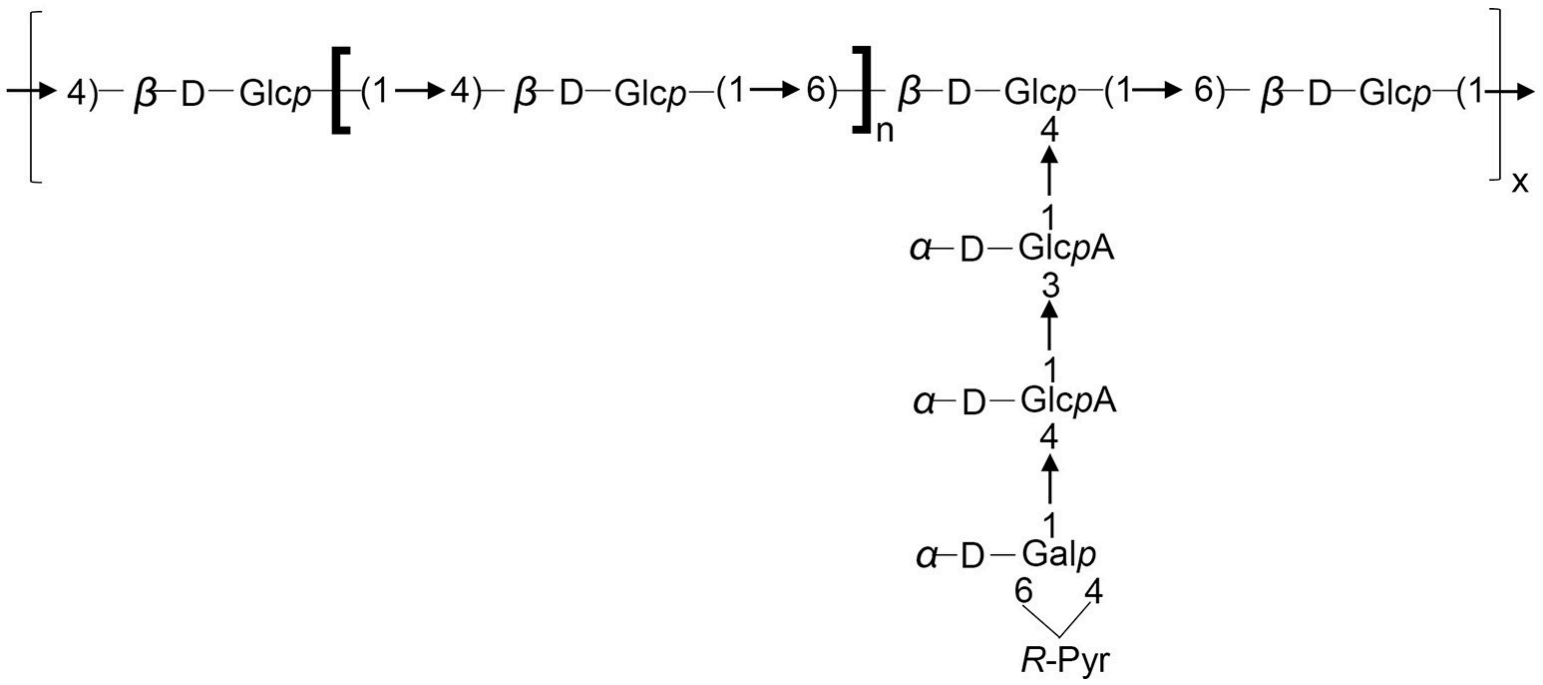
Structure of the repeating unit of the LMW EPS for *S. fredii* CCBAU45436 and *S. sojae* CCBAU05684. *n* = 2 or 3.

In summary, the atomic mass unit difference of 162 is representing one hexose (Glc or Gal) for the singly charged species (differences of 81 amu appear for the doubly charged ions). Such fragmentation pattern is related to sugar elimination from the non-reducing extremity of the saccharide, characteristic of Y ions. The four losses of 162 mass units. (Y8 m/z 1411.5 to Y5 m/z 925.3) are indicating that the non-reducing extremity have a length of 4 hexoses. Y8, Y7, and Y6 ions are undergoing losses of CO2 (−44 mass units loss for singly charged ions and −22 for the doubly charged) and pyruvic acid (-88 for singly charged ions and −44 for the doubly charged). The losses of 44 are related to the hexuronic acid moieties. The ion Yα8 (obtained by a loss of 232 = 162 + 88 – 18, for singly charged ion) indicated that the pyruvylated hexose is located on a second non-reducing end (Figure 5B). A side chain based on two uronic acids and a pyruvylated hexose is located on the penultimate position of the repeating unit stopping at the reducing end. The so obtained structure is completely suitable to the one established based on the NMR data resulting in the formula presented in Figure 6, which is composed of glucose, galactose, glucuronic acid, pyruvic acid in the ratios 5:1:2:1 or 6:1:2:1.

## Discussion

In this study, we aimed to determine whether the strains having different soybean cultivar host range release different symbiotic signals. We cultured four strains in laboratory condition and obtained their Nod factor structure using ESI-MS and ESI-MS/MS. The results showed that the four strains secreted common composition of Nod factors when induced by naringenin. They produced mostly tetramer (DP4) chitin backbones substituted by a methylfucose and a C18:1 fatty acid: Nod-IV (C18:1, Me-Fuc). The second most abundant metabolite was the Nod-V (C18:1, Me-Fuc), which is the corresponding pentamer. We did not find the N-methylation encoded by *nodS* nor the carbamoylation encoded by *nodU* or *nolO*. This could be explained by the fact that the truncated *nodSU* and *nolO* are not functional in these four strains. Similarly, *S. fredii* HH103 and *S. fredii* USDA257 have both truncated *nodSU* and *nolO* genes, and produce Nod factors structures that are not N-methylated nor carbamoyalated as discussed in previous studies (Madinabeitia et al., 2002; Vinardell et al., 2015). The sulfation is absent, indicating that *nodPQ* genes may not be expressed in the culture conditions tested. This explains why SJ05684 which doesn't have *nodPQ* released the same Nod factor structures. No significant differences could be observed among the compositions of the EPS produced by the four strains selected for this study. Moreover, we had only rare differences between the composition of the HMW and LMW EPS, except some enhanced proportions of mannose in the high molecular weight fraction. This is even more highlighted by comparing the ^1^H NMR profiles of all the samples, revealing a huge similarity. The mass spectrometric studies performed on all the produced EPS are close to identity. We describe for the first time the complete fragmentation of such acidic heterosaccharidic residue in the negative ionization mode. This allows us to give the exact number of sugars in the repeating unit and the localization of the side chain. We are reporting here that the representative structure of *S. fredii* or *S. sojae* EPS is composed of glucose, galactose, glucuronic acid, pyruvic acid in the ratios 5:1:2:1 or 6:1:2:1. Even if SJ05684 doesn't have *exoI*, it produced EPS that are identical to the other three strains. There may be some genes with similar functions in KPS or LPS clusters. All the findings above indicate that soybean cultivar compatibility of four strains is not dependent of Nod factor or EPS structure variety, although there are some differences in their gene clusters.

The here listed Nod factors are identical with those produced by *S. fredii* USDA257 or *S. fredii* HH103 (Bec Ferte et al., 1996; Gil-Serrano et al., 1997; Pueppke and Broughton, 1999). *B. diazoefficiens* USDA 110, a slow-growing soybean microsymbiont, produced one major Nod factor NodBj-V(C18:1, Me-Fuc) and a minor NodBj-V(Ac, C18:1, Me-Fuc) (Sanjuan et al., 1992; Carlson et al., 1993). Two other slow-growing strains, such as *B. japonicum* USDA135 and *B. elakanii* USDA61, produced various metabolites in addition to the USDA110 Nod factors (Table 4). *B. elakanii* USDA61 also produced four unique Nod metabolites where the reducing end bearing a fucose and a glycerol linked to the anomeric position, making it very different from other soybean microsymbionts. The structural differences among the Nod factor cocktails produced by these strains appeared to be involved in extending their host range. We tried to blast *nodA, nodB, nodC, nodZ*, and *noeI* nucleotides from different rhizobial strains that can nodulate soybean or coming from *Sinorhizobium* genus. The results showed that *nodB, nodC*, and *nodZ* genes of these strains are genus phylogenetic, but *nodA* and *noeI* of soybean microbionts were more closely related with each other (Figures S5, S6), indicating *nodA* or *noeI* is probably a key gene for soybean-rhizobia interaction. The *noeI* gene was reported to encode methylated fucose at the Nod factor reducing ends. Comparison analysis of Nod factor structures from different soybean microsymbionts or *Sinorhizobium* strains (summarized in Table 4) showed that Nod-V (C18:1, Me-Fuc) or at least its 2-O-methylfucose residue is a common structure or decoration, and thus seems to play a key role in the symbiosis between the rhizobia and soybean. Some molecular or genetic mutation studies have proved this point of view. The Nod-V (C18:1, Me-Fuc) secreted by *B. diazoefficiens* USDA110 was demonstrated as enabling deformation of the root hairs on *G. max*, but not as activating the response on the alfalfa roots (Sanjuan et al., 1992). *S. fredii* HH103 *noeI* mutant could still nodulate soybean, but its competitiveness was reduced (Madinabeitia et al., 2002). The nitrogen fixing ability of *B. diazoefficiens* USDA110 *noeI* mutant was degraded. Furthermore, single *noeI* or double *nodZnoeI mutant of S. fredii* CCBAU45436 showed decreasing nodule number, although their nitrogen fixing abilities were normal (Liu et al., 2017b). Although there are no specific genes shared by the soybean microsymbionts (Tian et al., 2012), all above indicates that rhizobial Nod factor compositions are much conserved, and that they are more related to host range than to phylogenetic relationship. Another example to support this conclusion is *Rhizobium* sp. IRBG74, which is the first known instance of a naturally occurring strain in this clade that is capable of forming nodules and fixing nitrogen with the legume. Both *Rhizobium* sp. IRBG74 and *Azorhizobium caulinodens* can nodulate *Sesbania cannabina* effectively, although their phylogenetic relationship is far from each other (Crook et al., 2013; Poinsot et al., 2016). Thus co-evolution is not the adapted concept. Actually, it is not straight to consider a specific co-evolution of the symbionts, but it is rather an adaptation of different bacteria to the selection pressure of the host plant. This could be related to the fact that rhizobia use probably a symbiotic pathway established between the endomyccorhizes and the plant, involving LCOs (Maillet et al., 2011; Liang et al., 2015).

**Table 4:** Summary of Nod metabolites structures from different rhizobial strains which can nodulate soybean or comes from *Sinorhizobium*.

**Strains**	**Acyl**	**R1**	**R2**	**R3**	**R4**	***n***	**Inducers**	**Symbiotic phenotype with soybean**	**References**
*S. fredii* CCBAU45436	C16:0, C16:1,C18:0, C18:1	H	H	H	Fuc, MeFuc	0, 1, 2	Naringenin	Nod+, Fix+	This study
*S. fredii* CCBAU25509	C16:0, C16:1, C18:0, C18:1	H	H	H	Fuc, MeFuc	0, 1, 2	Naringenin	Nod+, Fix+	This study
*S. fredii* HH103	C16:0, C16:1, C18:0,C18:1	H	H	H	Fuc, MeFuc	0, 1, 2	Genistein	Nod+, Fix+	Gil-Serrano et al., 1997
*S. fredii* USDA191^*^	C16:1, C18:0, C18:1	H	H	H	Fuc, MeFuc	0, 1, 2	Genistein	Nod+, Fix+	Bec Ferte et al., 1996
*S. fredii* USDA257	C18:1	H	H	H	Fuc, MeFuc	0, 1, 2	Genistein	Nod+, Fix+	Bec Ferte et al., 1996; Pueppke and Broughton, 1999
*S. sojae* CCBAU05684	C16:0, C16:1, C18:0, C18:1	H	H	H	Fuc, MeFuc	0, 1, 2	Naringenin	Nod+, Fix+	This study
*S*. sp. CCBAU05631	C16:0, C16:1, C18:0, C18:1	H	H	H	Fuc, MeFuc	0, 1, 2	Naringenin	Nod+, Fix+	This study
*S*. sp. NGR234	C16:1, C18:0, C18:1	Me	Cb, H	Cb, H	MeFuc, AcMeFuc, SMeFuc	2	Apigenin	Nod+, Fix-	Price et al., 1992; Pueppke and Broughton, 1999
*S. meliloti* RCR2011	C16:1, C16:2, C16:3	H	H	H, Ac	S	1, 2	Luteolin	Nod-, Fix-	Lerouge et al., 1990; Ardourel et al., 1994
*S. saheli* ORS611	C16:0, C18:1	Me	Cb	Cb	Fuc, H	2	Luteolin	Nod-, Fix-	Lorquin et al., 1997a
*S. teranga* bv. acaciae PRS1602	C16:0, C18:0, C18:1	Me	Cb	Cb	Fuc, H	2	Luteolin	Nod-, Fix-	Lorquin et al., 1997b
*B. elkanii* USDA61^**^	C18:1	Me, H	H	Ac, Cb, H	MeFuc	1, 2	Genistein	Nod+, Fix+	Carlson et al., 1993; Sanjuan et al., 1993
*B. diazoefficiens* USDA110	C18:1	H	H	Ac, H	MeFuc	2	Genistein, or soybean seed extract	Nod+, Fix+	Sanjuan et al., 1992; Carlson et al., 1993
*B. japonicum* USDA135	C16:0, C16:1,C18:1	H	H	Ac, H	MeFuc	2	Genistein	Nod^+^, Fix^+^	Sanjuan et al., 1993

*Ac, acetyl; Ara, arabinosyl; Cb, carbamoyl; Fuc, fucosyl; H, hydrogen; Me, methyl; S, sulfate; MeFuc, methylfucose; AcMeFuc, acetylated methylfucose; SMeFuc, sulfated methylfucose. R1, R2, R3, R4, R5 means different residues positioned on nod factor structures according to Figure 2A; n presents the number of polymerization. Nod±, the strain is/isn't able to nodulate soybean; Fix±, the strain is/isn't able to fix nitrogen with soybean*.

* *S. fredii USDA191 could produce a novel LCO with glucose substituted for GlcNAc in the backbone of the molecule*.

** *B. elkanii USDA61 could also produce four another Nod metabolites which are unique in that the reducing end N-acetylglucosamine contains a branching fucose and is glycosidically linked to glycerol: Nod-IV(C18:1, Fuc, Gro), Nod-IV(C18:1, Me, Fuc, Gro), Nod-IV(Cb, C18:1, Fuc, Gro), Nod-IV(Cb, C18:1, NMe, Fuc, Gro). Gro, Glycerol*.

The reported EPS structures in this study are similar to the ones published before for *S*. sp. NGR234 or *S. fredii* HH103. Exopolysaccharides secreted by *S*. sp. NGR234 or *S. fredii* HH103 strains are based on eight to nine saccharides within the repeating unit, which is composed of glucose, galactose and glucuronic acid, in a molar ratio 4:2:2 or 5:2:2, respectively. Both of them are substituted by a pyruvyl group (Djordjevic et al., 1986; Rodriguez-Navarro et al., 2014). However, some differences could be observed between the here studied exopolysaccharides and the ones reported in the literature: the strict absence of acetylation and of galactose on the linear backbone.

The EPS I synthesized by *S. meliloti* 1021 is composed of glucose and galactose in the 7:1 ratio, but is lacking the glucuronic acid. They are not only acetated and pyruvated, but succinylated. Besides, under phosphate starvation, *S. meliloti* can synthesize EPS II (galactoglucan), which is composed of repeated disaccharides without succinyl modifications (Zhan et al., 1991; Reinhold et al., 1994; Zevenhuizen, 1997). Even though the differences observed considering the non-carbohydrate residues, the EPSs saccharide backbones secreted by *Sinorhizobium* strains remain more similar than those obtained from *Bradyrhizobium*. The subunit of the exopolysaccharides synthesized by *B. diazoefficiens* USDA110 is made up of D-glucose, D-mannose, D-galacturonic acid and D-galactose in a molar ratio of 2:1:1:1, bearing 4-O-methylation and acetylations (Janczarek, 2011). EPS secreted by *B. elkanii* are composed of disaccharides made of L-rhamnose and 4-O-methyl-D-glucuronic acid in the 3:1 ratio (Minamisawa, 1989; An et al., 1995; Poveda et al., 1997). The structure comparison made above reinforces the fact that *Sinorhizobium* strains produce similar EPS compositions. Furthermore, the results indicate that EPS is more phylogenetic, probably because its main function is to help bacteria defend the biotic or abiotic stressed environment, in addition to be the symbiosis signal.

It was reported that KPS and T3SS in several soybean rhizobia are very important in determining host compatibility. In fact, the chemical structures of KPS are mostly strain specific. For example, there are clear differences between the composition of KPS in *S. fredii* strains nodulating only Asiatic or nodulating both Asiatic and American varieties of soybean. The firsts produce KPS with a sugar-Kdx repeating unit (Reuhs et al., 1993; Lopez-Baena et al., 2016), but the seconds release KPS do not follow the sugar-Kdx consensus (Reuhs et al., 1998; Gil-Serrano et al., 1999; Rodríguez-Carvajal et al., 2001, 2005; Margaret-Oliver et al., 2012). Besides, the wild type of *S. fredii* USDA257 could not induce effective nodules with American cultivars of soybean, however, many of its *tts* mutants unable to secret Nops gain the capacity to nodulate American soybeans (Meinhardt et al., 1993). Yet this is not always the same case. The wild type of *S. fredii* HH103 is able to induce Fix^+^ nodules on American soybean cultivars, even when this strain carries a functional symbiotic T3SS. Surpringly, the nodulation abilities of its *tts* mutants (*nopA, nopC*, and *nopX*) with American soybeans were reduced (Bellato et al., 1997; De Lyra et al., 2006; Lopez-Baena et al., 2008; Jiménez-Guerrero et al., 2015). Furthermore, recent research revealed that the mutation of genes encoding T3SS in three *Sinorhizobium* strains could change their cultivar symbiotic compatibility (Zhao et al., 2018). Therefore, T3SS may be involved in the capacity for rhizobia to nodulate soybean cultivars, and some of Nops may have different effects (detrimental or beneficial) in symbiosis depending on the specific couple of partners. All above indicates that the four strains in this study may have different structures of KPS, or special functions of nodulation proteins secreted by T3SS which results in the specific symbiotic compatibility. Their potential mechanisms remains to be elucidated in the future.

## Author contributions

VP contributed to the experimental designs, performed experiments, analyzed the results, and wrote the manuscript. DW performed the experiments, analyzed the results, and wrote the manuscript. FC analyzed the results, and wrote the manuscript. CT contributed to the experimental designs and revised the manuscript. WG and LL discussed and reviewed the manuscript.

### Conflict of interest statement

The authors declare that the research was conducted in the absence of any commercial or financial relationships that could be construed as a potential conflict of interest.
