# Supplementary material for: Conserved Composition of Nod Factors and Exopolysaccharides Produced by Different Phylogenetic Lineage Sinorhizobium Strains Nodulating Soybean
Source: Front Microbiol. 2018 Nov 26;9:2852. doi: 10.3389/fmicb.2018.02852 (PMC6275314; doi:10.3389/fmicb.2018.02852)
Supplement: Supplementary file 1 [file Data_Sheet_1.PDF]

TABLE S1

Summary of the 13C and 1H NMR chemical shifts measured for the LMW EPS of *Sinorhizobium fredii.**

	**Sugar**	**H1 C1 ppm**	**H2 C2 ppm**	**H3 C3 ppm**	**H4 C4 ppm**	**H5 C5 ppm**	**H6 C6 ppm**
A	→4) β-D Glc (1→ X2	4,45	3,25	3,57	3,71	3,58	3,89/3,77
		102,3	73	74,0	75,9	74.7	60,5
B	→4) β-D Glc (1→	4,48	3,38	3,57	3,68	3,64	3,84/3,67
		103,0	73	74,0	77,1	75,0	61,0
C	→ 6) β-D Glc (1→4	4,47	3,35	3,45	3,60	3,56	4,15/3,78
	↑	102,8	73	76,1	78,5	75,0	68,9
D	→ 6) β-D Glc (1→	4,45	3,26	3,41	3,71	3,60	4,15/3,63
		102,6	73	75,8	75,9	74,9	69,4
E	→3)-α-D GlcA 1→	5,32	3,64	3,75	3,55	3,95	/
		99,8	70,2	79	72,2	73,5	176,2
F	→ 4) -α-D GlcA 1→	5,30	3,67	3,90	3,56	4,25	/
		99,0	72,2	73,7	76,6	72,5	176,2
G	→ (4,6) α-D Gal 1→	5,43	3,84	3,66	4,10	3,73	3,91/3,78
		99,2	68,1	72,2	71,8	62,5	65,0
	Pyruvate	1.37	/	/			
		24.8	100.2	176.1			

* The shifts are given in ppm.

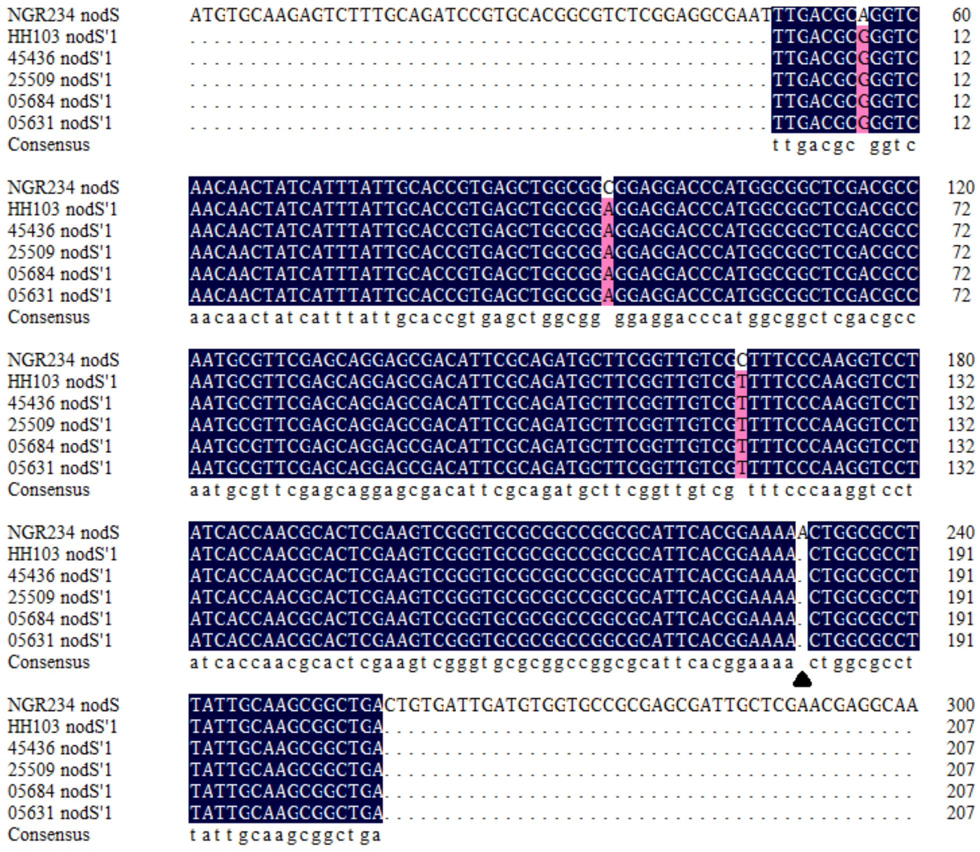

FIGURE S1. Alignment of DNA sequence containing the putative *nodS'1* regions of *Sinorhizobium fredii* strains (CCBAU45436, CCBAU2550, HH103), *S. sojae* CCBAU05684, *S.* sp. CCBAU05631, the *nodS* of *S.* sp. NGR234. The 1-bp deletions in *Sinorhizobium fredii* strains (CCBAU45436, CCBAU25509, HH103), *S. sojae* CCBAU05684, *S.* sp. CCBAU05631 sequences are indicated by black triangles.

```
NGR234 nodU    ATGCGCGTCTGTGGCATCAAGTTAACCCATGACGGGGCTATCGCCGTTGTCGAAGATGGA   60
HH103 nodU'1   ............................................................    0
45436 nodU'1   ............................................................    0
25509 nodU'1   ............................................................    0
05684 nodU'1   ............................................................    0
05631 nodU'1   ............................................................    0
Consensus

NGR234 nodU    AGGCTCGTCTTCTGTACTGAGCAGGAGAAGCGGAATAACAATTCGCGCTATCAAGAAATC  120
HH103 nodU'1   ............................................................    0
45436 nodU'1   ............................................................    0
25509 nodU'1   ............................................................    0
05684 nodU'1   ............................................................    0
05631 nodU'1   ............................................................    0
Consensus

NGR234 nodU    AACAATCTCGATGCTGTTGTGGCGGCTCTGGCGGAAAACGGGGTCAATGCACGAGACGTT  180
HH103 nodU'1   ..................GTGGCGGCTCTGGCGGAAAACGGGGTCAATGCAAGAGATGTT   42
45436 nodU'1   ..................GTGGCGGCTCTGGCGGAAAACGGGGTCAATGCAAGAGATGTT   42
25509 nodU'1   ..................GTGGCGGCTCTGGCGGAAAACGGGGTCAATGCAAGAGATGTT   42
05684 nodU'1   ..................GTGGCGGCTCTGGCGGAAAACGGGGTCAATGCAAGAGATGTT   42
05631 nodU'1   ..................GTGGCGGCTCTGGCGGAAAACGGGGTCAATGCAAGAGATGTT   42
Consensus                        gtggcggctctggcggaaaacggggtcaatgca gaga gtt

NGR234 nodU    GATCAGTTTGTCATCGACGGCTGGGACGGTGAGGCCGAGTCGCAGTTCAAGGTCCTCAGT  240
HH103 nodU'1   GATCAGTTTGTCATCGACGGCTGGGACGGTGAGGCCGAGTCGCGGTTCAAGGTCCTCAGT  102
45436 nodU'1   GATCAGTTTGTCATCGACGGCTGGGACGGTGAGGCCGAGTCGCGGTTCAAGGTCCTCAGT  102
25509 nodU'1   GATCAGTTTGTCATCGACGGCTGGGACGGTGAGGCCGAGTCGCGGTTCAAGGTCCTCAGT  102
05684 nodU'1   GATCAGTTTGTCATCGACGGCTGGGACGGTGAGGCCGAGTCGCGGTTCAAGGTCCTCAGT  102
05631 nodU'1   GATCAGTTTGTCATCGACGGCTGGGACGGTGAGGCCGAGTCGCGGTTCAAGGTCCTCAGT  102
Consensus      gatcagtttgtcatcgacggctgggacggtgaggccgagtcgc gttcaaggtcctcagt

NGR234 nodU    GGGGAGACTCCTGTTATCCTCAGAGGCGCGCCTTACGTTGAACGCCACGCCGAGGGACTT  300
HH103 nodU'1   GGGGAGACTCCTGTTATCCTCAGAGGCGCGCCTTACGTTGAACGTCACGCCGAGGGACTT  162
45436 nodU'1   GGGGAGACTCCTGTTATCCTCAGAGGCGCGCCTTACGTTGAACGTCACGCCGAGGGACTT  162
25509 nodU'1   GGGGAGACTCCTGTTATCCTCAGAGGCGCGCCTTACGTTGAACGTCACGCCGAGGGACTT  162
05684 nodU'1   GGGGAGACTCCTGTTATCCTCAGAGGCGCGCCTTACGTTGAACGTCACGCCGAGGGACTT  162
05631 nodU'1   GGGGAGACTCCTGTTATCCTCAGAGGCGCGCCTTACGTTGAACGTCACGCCGAGGGACTT  162
Consensus      ggggagactcctgttatcctcagaggcgcgccttacgttgaacg cacgccgagggactt

NGR234 nodU    CTCGATTGGATCGGCGGCTCCGGCCTCACACTTGGTGATCGGGTTTTTAGCTACAGAAGC  360
HH103 nodU'1   CTCGATTGGATCGGCGGCTCCGGCCTCACACTTGGTGATCGGGTTTTTAGCTACAGAAGC  222
45436 nodU'1   CTCGATTGGATCGGCGGCTCCGGCCTCACACTTGGTGATCGGGTTTTTAGCTACAGAAGC  222
25509 nodU'1   CTCGATTGGATCGGCGGCTCCGGCCTCACACTTGGTGATCGGGTTTTTAGCTACAGAAGC  222
05684 nodU'1   CTCGATTGGATCGGCGGCTCCGGCCTCACACTTGGTGATCGGGTTTTTAGCTACAGAAGC  222
05631 nodU'1   CTCGATTGGATCGGCGGCTCCGGCCTCACACTTGGTGATCGGGTTTTTAGCTACAGAAGC  222
Consensus      ctcgattggatcggcggctccggcctcacacttggtgatcgggtttttagctacagaagc

NGR234 nodU    TATCCGCATGTTACGAGCCATGTCGCCTCTGCATACTGCACCAGCCCCTTTGCCAAATCC  420
HH103 nodU'1   TATCCGCATGTGACGAGCCATGTCGCCTCTGCATACTGCACCAGCCCCTTTGCCAAATCC  282
45436 nodU'1   TATCCGCATGTGACGAGCCATGTCGCCTCTGCATACTGCACCAGCCCCTTTGCCAAATCC  282
25509 nodU'1   TATCCGCATGTGACGAGCCATGTCGCCTCTGCATACTGCACCAGCCCCTTTGCCAAATCC  282
05684 nodU'1   TATCCGCATGTGACGAGCCATGTCGCCTCTGCATACTGCACCAGCCCCTTTGCCAAATCC  282
05631 nodU'1   TATCCGCATGTGACGAGCCATGTCGCCTCTGCATACTGCACCAGCCCCTTTGCCAAATCC  282
Consensus      tatccgcatgt acgagccatgtcgcctctgcatactgcaccagcccctttgccaaatcc

NGR234 nodU    GGAGACCCTGCGCTTTGCCTGGTGTGGGACGGCTGCATATTTCCGGGGCTCTACCATGTG  480
HH103 nodU'1   GGAGACCCTGCGCTTTGCCTGGTGTGGGACGGCTGCATATTTCCGCAGCTCTACCATGTG  342
45436 nodU'1   GGAGACCCTGCGCTTTGCCTGGTGTGGGACGGCTGCATATTTCCGCAGCTCTACCATGTG  342
25509 nodU'1   GGAGACCCTGCGCTTTGCCTGGTGTGGGACGGCTGCATATTTCCGCAGCTCTACCATGTG  342
05684 nodU'1   GGAGACCCTGCGCTTTGCCTGGTGTGGGACGGCTGCATATTTCCGCAGCTCTACCATGTG  342
05631 nodU'1   GGAGACCCTGCGCTTTGCCTGGTGTGGGACGGCTGCATATTTCCGCGGCTCTACCATGTG  342
Consensus      ggagaccctgcgctttgcctggtgtgggacggctgcatatttccgc gctctaccatgtg

NGR234 nodU    GAAGGCAAGCGAGCCAGCTTCGTCAAATCGTTGTTCCCGGTAACAGGCCAGGCCTACGCT  540
HH103 nodU'1   GAAGGCAAGCGAGCCAGCTTCGTCAAATCCTTGTTCCCGGTAACAGGTCAGGCCTACGCT  402
45436 nodU'1   GAAGGCAAGCGAGCCAGCTTCGTCAAATCCTTGTTCCCGGTAACAGGTCAGGCCTACGCT  402
25509 nodU'1   GAAGGCAAGCGAGCCAGCTTCGTCAAATCCTTGTTCCCGGTAACAGGTCAGGCCTACGCT  402
05684 nodU'1   GAAGGCAAGCGAGCCAGCTTCGTCAAATCCTTGTTCCCGGTAACAGGTCAGGCCTACGCT  402
05631 nodU'1   GAAGGCAAGCGAGCCAGCTTCGTCAAATCCTTGTTCCCGGTAACAGGTCAGGCCTACGCT  402
Consensus      gaaggcaagcgagccagcttcgtcaaatc ttgttcccggtaacagg caggcctacgct

NGR234 nodU    GCCGCGGGCCACTACTTCGGCCCCTATAAGCAGACGAGCCGCGGGGGCTGGGACCTCGGC  600
HH103 nodU'1   GCCGCGGGCCACTACTTCGGCCCCTATAAGCAGACGAGCCGCGGGGGCTGGGACCTTGGC  462
45436 nodU'1   GCCGCGGGCCACTACTTCGGCCCCTATAAGCAGACGAGCCGCGGGGGCTGGGACCTTGGC  462
25509 nodU'1   GCCGCGGGCCACTACTTCGGCCCCTATAAGCAGACGAGCCGCGGGGGCTGGGACCTTGGC  462
05684 nodU'1   GCCGCGGGCCACTACTTCGGCCCCTATAAGCAGACGAGCCGCGGGGGCTGGGACCTTGGC  462
05631 nodU'1   GCCGCGGGCCACTACTTCGGCCCCTATAAGCAGACGAGCCGCGGGGGCTGGGACCTCGGC  462
Consensus      gccgcgggccactacttcggcccctataagcagacgagccgcgggggctgggacct ggc

NGR234 nodU    GTTGCCGGCAAGCTGATGGCCTTTATCGCACTGGGATCAGTTCACGAACGCATCGTTGCT  660
HH103 nodU'1   GTTGCCGGCAAACTGATGGCCTTTATCGCACTGGGATCAGTTCACGTACGCATCGTTGCT  522
45436 nodU'1   GTTGCCGGCAAACTGATGGCCTTTATCGCACTGGGATCAGTTCACGTACGCATCGTTGCT  522
25509 nodU'1   GTTGCCGGCAAACTGATGGCCTTTATCGCACTGGGATCAGTTCACGTACGCATCGTTGCT  522
05684 nodU'1   GTTGCCGGCAAACTGATGGCCTTTATCGCACTGGGATCAGTTCACGTACGCATCGTTGCT  522
05631 nodU'1   GTTGCCGGCAAACTGATGGCCTTTATCGCACCGGGATCAGTTCACGTACGCATCGTTGCT  522
Consensus      gttgccggcaa ctgatggcctttatcgcac gggatcagttcacg acgcatcgttgct
```

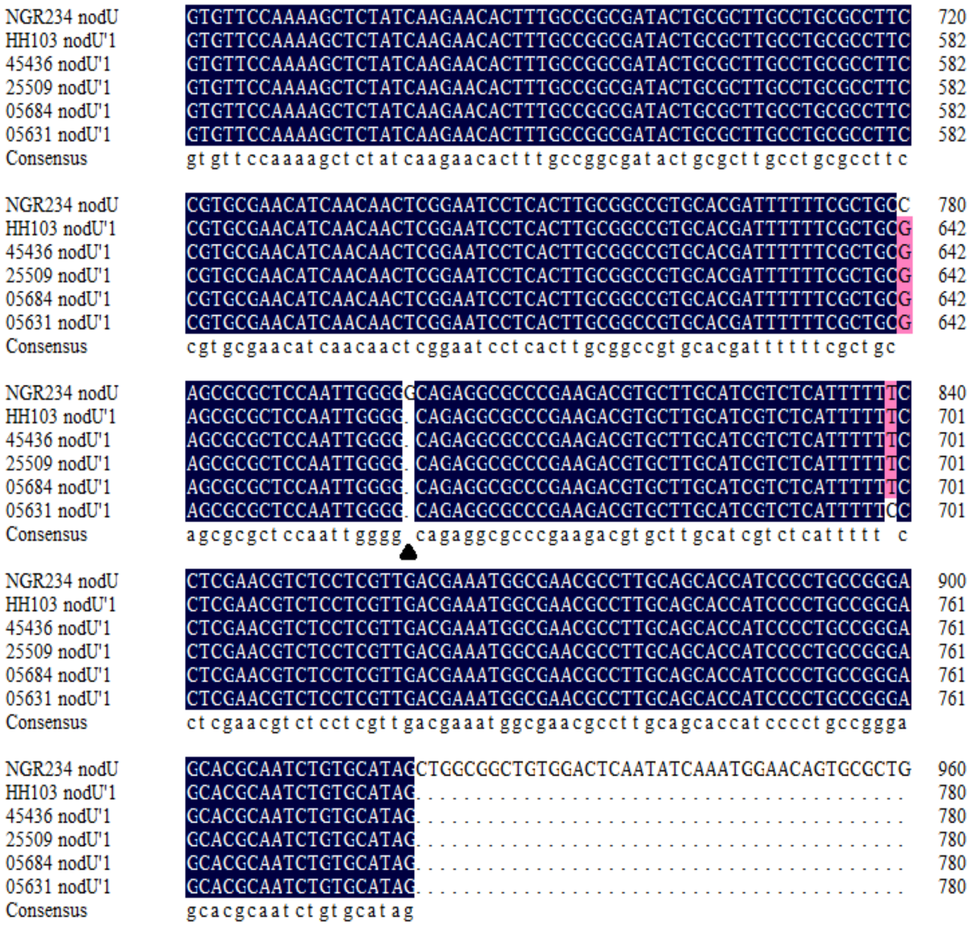

FIGURE S2. Alignment of DNA sequence containing the putative *nodU'1* regions of *Sinorhizobium fredii* strains (CCBAU45436, CCBAU25509, HH103), *S. sojae* CCBAU05684, *S.* sp. CCBAU05631, the *nodU* of *S.* sp. NGR234. The 1-bp deletions in *Sinorhizobium fredii* strains (CCBAU45436, CCBAU25509, HH103), *S. sojae* CCBAU05684, *S.* sp. CCBAU05631 sequences are indicated by black triangles.

```
NGR234 nolO      ATGCTGTGTCTAGGACTTAGTGGCGGTCTAAGCAAGATTCACGAAAACTCGCTCGATCTG   60
HH103 nolO HNU   ATGCTGTGTCTAGGACTGAGTGGCGGTCTAAGCAAGATTTACGAAAACACGCTCGATCTG   60
45436 nolO HNU   ATGCTGTGTCTAGGACTGAGTGGCGGTCTAAGCAAGATTTACGAAAACACGCTCGATCTG   60
25509 nolO HNU   ATGCTGTGTCTAGGACTGAGTGGCGGTCTAAGCAAGATTTACGAAAACACGCTCGATCTG   60
05684 nolO HNU   ATGCTGTGTCTAGGACTGAGTGGCGGTCTAAGCAAGATTTACGAAAACACGCTCGATCTG   60
05631 nolO HNU   ATGCTGTGTCTAGGACTGAGTGGCGGTCTAAGCAGGATTTACGAAAACTCGCTCGATCTG   60
Consensus        atgctgtgtctaggact agtggcggtctaagca gatt acgaaaac cgctcgatctg

NGR234 nolO      CCGAATACATTTATGCACGACGGTGCAGCGGTTCTCGTCCGAGACGGCCAAGTGATAGCT   120
HH103 nolO HNU   CCGAATACATTTATGCACGACGGTGCAGCGGTTCTCGTCCGAGACGGCCGAGTGATAGCT   120
45436 nolO HNU   CCGAATACATTTATGCACGACGGTGCAGCGGTTCTCGTCCGAGACGGCCGAGTGATAGCT   120
25509 nolO HNU   CCGAATACATTTATGCACGACGGTGCAGCGGTTCTCGTCCGAGACGGCCGAGTGATAGCT   120
05684 nolO HNU   CCGAATACATTTATGCACGACGGTGCAGCGGTTCTCGTCCGAGACGGCCGAGTGATAGCT   120
05631 nolO HNU   CCGAATACATTTATGCACGACGGTGCAGCGGTTCTCGTCCGAGACGGCCGAGTGATAGCT   120
Consensus        ccgaatacatttatgcacgacggtgcagcggttctcgtccgagacggcc agtgatagct

NGR234 nolO      GCTGTCGAAGAGGAGCGACTTAACAGAATCAAACATTCCAACAAGCTGCCGCGCCGGTCG   180
HH103 nolO HNU   GCTGTCGAAGAGGAGCGGCTTAATAGAATCAAACATTCCAACAAGCTGCCGCGCCGTTCG   180
45436 nolO HNU   GCTGTCGAAGAGGAGCGGCTTAATAGAATCAAACATTCCAACAAGCTGCCGCGCCGTTCG   180
25509 nolO HNU   GCTGTCGAAGAGGAGCGGCTTAATAGAATCAAACATTCCAACAAGCTGCCGCGCCGTTCG   180
05684 nolO HNU   GCTGTCGAAGAGGAGCGGCTTAATAGAATCAAACATTCCAACAAGCTGCCGCGCCGTTCG   180
05631 nolO HNU   GCTGTCGAAGAGGAGCGGCTTAATAGAATCAAACATTCCAACAAGCTGCCGCGCCGTTCG   180
Consensus        gctgtcgaagaggagcg cttaa agaatcaaacattccaacaagctgccgcgccg tcg

NGR234 nolO      ATTCAATACTGCCTTGAATACGCCGGGGTTCAACTCAGCGACATCGACTGCATCGCGTAT   240
HH103 nolO HNU   ATTCAATACTGCCTTGAATACGCCGGGGTTCAGCTCAGCGACATCGACTGC.TCGCGTAT   239
45436 nolO HNU   ATTCAATACTGCCTTGAATACGCCGGGGTTCAGCTCAGCGACATCGACTGC.TCGCGTAT   239
25509 nolO HNU   ATTCAATACTGCCTTGAATACGCCGGGGTTCAGCTCAGCGACATCGACTGC.TCGCGTAT   239
05684 nolO HNU   ATTCAATACTGCCTTGAATACGCCGGGGTTCAGCTCAGCGACATCGACTGC.TCGCGTAT   239
05631 nolO HNU   ATTCAATACTGCCTTGAATACGCCGGGGTTCAGCTCAGCGACATCGACTGC.TCGCGTAT   239
Consensus        attcaatactgccttgaatacgccggggttca ctcagcgacatcgactgc tcgcgtat
                                                                    ▲

NGR234 nolO      TATGCAACCGAGGCGTTTTGCAATGCTATGCTCGAGCGCCTGCTCGTTTCTCAGTCGCAC   300
HH103 nolO HNU   TATGCAACCGAGGCATTTTGCAATGCTATGCTCGAGCGCTTGCTCGTTTCTCAGCCGCAC   299
45436 nolO HNU   TATGCAACCGAGGCATTTTGCAATGCTATGCTCGAGCGCTTGCTCGTTTCTCAGCCGCAC   299
25509 nolO HNU   TATGCAACCGAGGCATTTTGCAATGCTATGCTCGAGCGCTTGCTCGTTTCTCAGCCGCAC   299
05684 nolO HNU   TATGCAACCGAGGCATTTTGCAATGCTATGCTCGAGCGCTTGCTCGTTTCTCAGCCGCAC   299
05631 nolO HNU   TATGCAACCGAGGCATTTTGCAATGCTATGCTCGAGCGCTTGCTCGTTTCTCAGCCGCAC   299
Consensus        tatgcaaccgaggc ttttgcaatgctatgctcgagcgc tgctcgtttctcag cgcac

NGR234 nolO      ATGTCAATACCGTTGGATGCCAAGCTGTTGTTGCGTGGGTTGCTCGCGCAGGAATTTGGT   360
HH103 nolO HNU   ATGTCAATACCGTTGTATGCCAAGCTGTTGTTGTGTGGGTTGCTCGCGCAGGAATTTGGT   359
45436 nolO HNU   ATGTCAATACCGTTGTATGCCAAGCTGTTGTTGTGTGGGTTGCTCGCGCAGGAATTTGGT   359
25509 nolO HNU   ATGTCAATACCGTTGTATGCCAAGCTGTTGTTGTGTGGGTTGCTCGCGCAGGAATTTGGT   359
05684 nolO HNU   ATGTCAATACCGTTGTATGCCAAGCTGTTGTTGTGTGGGTTGCTCGCGCAGGAATTTGGT   359
05631 nolO HNU   ATGTCAATACCGTTGAATGCCAAGCTGTTGTTGTGTGGGTTGCTCGCGCAGGAATTTGGT   359
Consensus        atgtcaataccgttg atgccaagctgttgttg gtgggttgctcgcgcaggaatttggt

NGR234 nolO      ACTGAGGTCGATCCGTCGCGAATCTCATTCGTAAGCCATCACCTGTCGCACGCCTGGAGC   420
HH103 nolO HNU   ACTGAGGTCGATCCGTCGCGAATCTCATTCGTAAGCCATCACCTGTCGCACGCCTGGAGC   419
45436 nolO HNU   ACTGAGGTCGATCCGTCGCGAATCTCATTCGTAAGCCATCACCTGTCGCACGCCTGGAGC   419
25509 nolO HNU   ACTGAGGTCGATCCGTCGCGAATCTCATTCGTAAGCCATCACCTGTCGCACGCCTGGAGC   419
05684 nolO HNU   ACTGAGGTCGATCCGTCGCGAATCTCATTCGTAAGCCATCACCTGTCGCACGCCTGGAGC   419
05631 nolO HNU   ACTGAGGTCGATCCGTCGCGAATCTCATTCGTAAGCCATCACCTGTCGCACGCCTGGAGC   419
Consensus        actgaggtcgatccgtcgcgaatctcattcgtaagccatcacctgtcgcacgcctggagc

NGR234 nolO      GCGTTTTCTATGTCTGGTTTCGAACAAAGTCTCATCTTGACAATTGATGGCGGTGGAGAC   480
HH103 nolO HNU   GCGTTTTCTATGTCGGGTTTCGAACAAAGTCTCATCTTGACAATTGATGGCGGTGGAGAC   479
45436 nolO HNU   GCGTTTTCTATGTCGGGTTTCGAACAAAGTCTCATCTTGACAATTGATGGCGGTGGAGAC   479
25509 nolO HNU   GCGTTTTCTATGTCGGGTTTCGAACAAAGTCTCATCTTGACAATTGATGGCGGTGGAGAC   479
05684 nolO HNU   GCGTTTTCTATGTCGGGTTTCGAACAAAGTCTCATCTTGACAATTGATGGCGGTGGAGAC   479
05631 nolO HNU   GCGTTTTCTATGTCGGGTTTCGAACAAAGTCTCATCTTGACAATTGATGGCGGTGGAGAC   479
Consensus        gcgttttctatgtc ggtttcgaacaaagtctcatcttgacaattgatggcggtggagac

NGR234 nolO      TTCGCCTCGGGTCTTTTGGCGGTAGGATCCGGCACGGAAGTCAAACCGCTTGCGACATTT   540
HH103 nolO HNU   TTCGCCTCGGGTCTTTTGGCGATAGGATCTGGCACGGAAGTCACACCGCTTGCGACATTT   539
45436 nolO HNU   TTCGCCTCGGGTCTTTTGGCGATAGGATCTGGCACGGAAGTCACACCGCTTGCGACATTT   539
25509 nolO HNU   TTCGCCTCGGGTCTTTTGGCGATAGGATCTGGCACGGAAGTCACACCGCTTGCGACATTT   539
05684 nolO HNU   TTCGCCTCGGGTCTTTTGGCGATAGGATCTGGCACGGAAGTCACACCGCTTGCGACATTT   539
05631 nolO HNU   TTCGCCTCGGGTCTTTTGGCGGTAGGATCTGGCACGGAAGTCACACCGCTTGCGACATTT   539
Consensus        ttcgcctcgggtcttttggcg taggatc ggcacggaagtca accgcttgcgacattt

NGR234 nolO      CCGGAGAGCGATTCTTTAGGGCTTTTGTACCTTGAGACCATAAAATATCTCGGTTACGGC   600
HH103 nolO HNU   CCGGAGAGCGATTCTTTAGGGCTTTTGTACCTTGAGACCATAAAATATCTCGGTTACGGC   599
45436 nolO HNU   CCGGAGAGCGATTCTTTAGGGCTTTTGTACCTTGAGACCATAAAATATCTCGGTTACGGC   599
25509 nolO HNU   CCGGAGAGCGATTCTTTAGGGCTTTTGTACCTTGAGACCATAAAATATCTCGGTTACGGC   599
05684 nolO HNU   CCGGAGAGCGATTCTTTAGGGCTTTTGTACCTTGAGACCATAAAATATCTCGGTTACGGC   599
05631 nolO HNU   CCGGAGAGCGATTCTTTAGGGCTTCTGTACCTTGAGACCATAAAATATCTCGGTTACGGC   599
Consensus        ccggagagcgattctttagggctt tgtaccttgagaccataaaatatctcggttacggc

NGR234 nolO      ATGTTTGATGAATACAAGGTCATGGGGCTTGCACCGTACGGCGACCCCGCTCCCCATCGC   660
HH103 nolO HNU   ATGTTTGATGAATACAAGGTCATGGGGCTTGCACCGTACGGCGACCCCGCTCCCCATCGC   659
45436 nolO HNU   ATGTTTGATGAATACAAGGTCATGGGGCTTGCACCGTACGGCGACCCCGCTCCCCATCGC   659
25509 nolO HNU   ATGTTTGATGAATACAAGGTCATGGGGCTTGCACCGTACGGCGACCCCGCTCCCCATCGC   659
05684 nolO HNU   ATGTTTGATGAATACAAGGTCATGGGGCTTGCACCGTACGGCGACCCCGCTCCCCATCGC   659
05631 nolO HNU   ATGTTTGATGAATACAAGGTCATGGGGCTTGCACCGTACGGCGACCCCGCTCCCCATCGC   659
Consensus        atgtttgatgaatacaaggtcatggggcttgcaccgtacggcgaccccgctccccatcgc
```

```
NGR234 nolO       GACCTCTTCGAACAGTTCTACGAGCTATTAGACAACGGTGGCTACCGGATCTATCTGGAC   720
HH103 nolO HNU    GACCTCTTCGAACAGTTCTACGAGCTATTAGACAACGGTGGCTACCGGATCTATCTGGAC   719
45436 nolO HNU    GACCTCTTCGAACAGTTCTACGAGCTATTAGACAACGGTGGCTACCGGATCTATCTGGAC   719
25509 nolO HNU    GACCTCTTCGAACAGTTCTACGAGCTATTAGACAACGGTGGCTACCGGATCTATCTGGAC   719
05684 nolO HNU    GACCTCTTCGAACAGTTCTACGAGCTATTAGACAACGGTGGCTACCGGATCTATCTGGAC   719
05631 nolO HNU    GACCTCTTCGAACAGTTCTACGAGCTATTAGACAACGGTGGCTACCGGATCTATCTGGAC   719
Consensus         gacctcttcgaacagttctacgagctattagacaacggtggctaccggatctatctggac

NGR234 nolO       CGGATTGGTCCCACGCTGCTTCGCAGCATTGAGGTACGCCGAAAAGGAATGCCGTTCACT   780
HH103 nolO HNU    CGGATTGGTCCCACGCTGCTTCGCAGCATTGAGGTACGCCGAAAAGGGATGCCGTTCACT   779
45436 nolO HNU    CGGATTGGTCCCACGCTGCTTCGCAGCATTGAGGTACGCCGAAAAGGGATGCCGTTCACT   779
25509 nolO HNU    CGGATTGGTCCCACGCTGCTTCGCAGCATTGAGGTACGCCGAAAAGGGATGCCGTTCACT   779
05684 nolO HNU    CGGATTGGTCCCACGCTGCTTCGCAGCATTGAGGTACGCCGAAAAGGGATGCCGTTCACT   779
05631 nolO HNU    CGGATTGGTCCCACGCTGCTTCGCAGCATTGAGGTACGCCGAAAAGGGATGCCGTTCACT   779
Consensus         cggattggtcccacgctgcttcgcagcattgaggtacgccgaaaagg atgccgttcact

NGR234 nolO       CAGCAGCATAAAGATTTAAGTGCTTCGTTGCAAGAAGCACTCGAACGGATTGTGTTTCAC   840
HH103 nolO HNU    CAGCAGCATAAAGATTTCAGTGCTTCGTTGCAAGAAGCACTCGAACGGATTGTGTTTCAC   839
45436 nolO HNU    CAGCAGCATAAAGATTTCAGTGCTTCGTTGCAAGAAGCACTCGAACGGATTGTGTTTCAC   839
25509 nolO HNU    CAGCAGCATAAAGATTTCAGTGCTTCGTTGCAAGAAGCACTCGAACGGATTGTGTTTCAC   839
05684 nolO HNU    CAGCAGCATAAAGATTTCAGTGCTTCGTTGCAAGAAGCACTCGAACGGATTGTGTTTCAC   839
05631 nolO HNU    CAGCAGCATAAAGATTTCAGTGCTTCGTTGCAAGAAGCACTCGAACGGATTGTGTTTCAC   839
Consensus         cagcagcataaagattt agtgcttcgttgcaagaagcactcgaacggattgtgtttcac

NGR234 nolO       GTTCTACGGCATCACAGCGAGATCACCGGCATAAAGCGCTTGAGCTTGGCCGGAGGAGTA   900
HH103 nolO HNU    GTTCTACGGCATCACAGCGAGATCACCGGCATAAAGCGCTTAAGCTTGGCCGGAGGAGTA   899
45436 nolO HNU    GTTCTACGGCATCACAGCGAGATCACCGGCATAAAGCGCTTAAGCTTGGCCGGAGGAGTA   899
25509 nolO HNU    GTTCTACGGCATCACAGCGAGATCACCGGCATAAAGCGCTTAAGCTTGGCCGGAGGAGTA   899
05684 nolO HNU    GTTCTACGGCATCACAGCGAGATCACCGGCATAAAGCGCTTAAGCTTGGCCGGAGGAGTA   899
05631 nolO HNU    GTTCTACGGCATCACAGCGAGATCACCGGCATCAAGCGCTTAAGCTTGGCCGGAGGAGTA   899
Consensus         gttctacggcatcacagcgagatcaccggcat aagcgctt agcttggccggaggagta

NGR234 nolO       GCTCACAACTGCACATTGAACGGCAAGCTGTTGCGCTCGGGAATCTTCCAAGACATCTTC   960
HH103 nolO HNU    GCTCACAACTGCACTTTGAGCGGTAAGCTGTTGCGCTCGGGAATCTTCCAAGACATCTTC   959
45436 nolO HNU    GCTCACAACTGCACTTTGAGCGGTAAGCTGTTGCGCTCGGGAATCTTCCAAGACATCTTC   959
25509 nolO HNU    GCTCACAACTGCACTTTGAGCGGTAAGCTGTTGCGCTCGGGAATCTTCCAAGACATCTTC   959
05684 nolO HNU    GCTCACAACTGCACTTTGAGCGGTAAGCTGTTGCGCTCGGGAATCTTCCAAGACATCTTC   959
05631 nolO HNU    GCTCACAACTGCACTTTGAACGGTAAGCTGTTGCGCTCGGGAATCTTCCAAGACATCTTC   959
Consensus         gctcacaactgcac ttga cgg aagctgttgcgctcgggaatcttccaagacatcttc

NGR234 nolO       GTGCAACCCGCGGCACACGACGCTGGCTGCGCATTAGGCGCTGCATTGATGATGTCTAAT   1020
HH103 nolO HNU    GTGCAACCCGCGGCACACGACGCTGGCTGCGCATTAGGCGCTGCATTGATGATGTCTAAT   1019
45436 nolO HNU    GTGCAACCCGCGGCACACGACGCTGGCTGCGCATTAGGCGCTGCATTGATGATGTCTAAT   1019
25509 nolO HNU    GTGCAACCCGCGGCACACGACGCTGGCTGCGCATTAGGCGCTGCATTGATGATGTCTAAT   1019
05684 nolO HNU    GTGCAACCCGCGGCACACGACGCTGGCTGCGCATTAGGCGCTGCATTGATGATGTCTAAT   1019
05631 nolO HNU    GTGCAACCCGCGGCACACGACGCTGGCTGCGCATTAGGAGCTGCATTGATGATGTCTAAT   1019
Consensus         gtgcaacccgcggcacacgacgctggctgcgcattagg gctgcattgatgatgtctaat

NGR234 nolO       GAACTAGGGCAGTCCGCACCTCGGGAGCGTTTGCAGGAGGTCTATTGGGGTCCTGATCTC   1080
HH103 nolO HNU    GAACTAGGGCAGTCCGCACCTCGAGAGCGTTTGCAGGAGGTCTATTGGGGTCCTGATCTC   1079
45436 nolO HNU    GAACTAGGGCAGTCCGCACCTCGAGAGCGTTTGCAGGAGGTCTATTGGGGTCCTGATCTC   1079
25509 nolO HNU    GAACTAGGGCAGTCCGCACCTCGAGAGCGTTTGCAGGAGGTCTATTGGGGTCCTGATCTC   1079
05684 nolO HNU    GAACTAGGGCAGTCCGCACCTCGAGAGCGTTTGCAGGAGGTCTATTGGGGTCCTGATCTC   1079
05631 nolO HNU    GAACTAGGGCAGTCCGCACCTCGAGAGCGTTTGCAGGAGGTCTATTGGGGTCCTGATCTC   1079
Consensus         gaactagggcagtccgcacctcg gagcgtttgcaggaggtctattggggtcctgatctc

NGR234 nolO       GGGAGCGACCGCGCCGTGGAGCAGGAATTAATAGCATGGGGCGGCCACATTGAAATCGAA   1140
HH103 nolO HNU    GGGAGCGACCGCGCCGTGGAGCAGGAATTAATTGCATGGGGCGGCCACATTGAAATCGAA   1139
45436 nolO HNU    GGGAGCGACCGCGCCGTGGAGCAGGAATTAATTGCATGGGGCGGCCACATTGAAATCGAA   1139
25509 nolO HNU    GGGAGCGACCGCGCCGTGGAGCAGGAATTAATTGCATGGGGCGGCCACATTGAAATCGAA   1139
05684 nolO HNU    GGGAGCGACCGCGCCGTGGAGCAGGAATTAATTGCATGGGGCGGCCACATTGAAATCGAA   1139
05631 nolO HNU    GGGAGCGACCGCGCCGTGGAGCAGGAATTAATTGCATGGGGCGGCCACATTGAAATCGAA   1139
Consensus         gggagcgaccgcgccgtggagcaggaattaat gcatggggcggccacattgaaatcgaa

NGR234 nolO       CGCTGCGATGATGTGGCCAGCAGAGCAGCCGAGTGGATCGCCCATGGCGCCGTGATCGGC   1200
HH103 nolO HNU    CGCTGCGATGATGTGGCCAGCAGAGCAGCCGAGTGGATCGCCCATGGCGCCGTTATCGGC   1199
45436 nolO HNU    CGCTGCGATGATGTGGCCAGCAGAGCAGCCGAGTGGATCGCCCATGGCGCCGTTATCGGC   1199
25509 nolO HNU    CGCTGCGATGATGTGGCCAGCAGAGCAGCCGAGTGGATCGCCCATGGCGCCGTTATCGGC   1199
05684 nolO HNU    CGCTGCGATGATGTGGCCAGCAGAGCAGCCGAGTGGATCGCCCATGGCGCCGTTATCGGC   1199
05631 nolO HNU    CGCTGCGATGATGTGGCCAGCAGAGCAGCCGAGTGGATCGCC.ATGGCGCCGTTATCGGC   1198
Consensus         cgctgcgatgatgtggccagcagagcagccgagtggatcgcc atggcgccgt atcggc

NGR234 nolO       TGGGTGCAGGGACGATCGGAATTCGGACCGCGTGCGCTTGGCAACCGTAGCATTCTTGCC   1260
HH103 nolO HNU    TGGATGCAGGGACGATCGGAATTCGGACCGCGTGCGCTTGGCAACCGTAGCATTCTTGCC   1259
45436 nolO HNU    TGGATGCAGGGACGATCGGAATTCGGACCGCGTGCGCTTGGCAACCGTAGCATTCTTGCC   1259
25509 nolO HNU    TGGATGCAGGGACGATCGGAATTCGGACCGCGTGCGCTTGGCAACCGTAGCATTCTTGCC   1259
05684 nolO HNU    TGGATGCAGGGACGATCGGAATTCGGACCGCGTGCGCTTGGCAACCGTAGCATTCTTGCC   1259
05631 nolO HNU    TGGATGCAGGGACGATCGGAATTCGGACCGCGTGCGCTTGGCAACCGTAGCATTCTTGCC   1258
Consensus         tgg tgcagggacgatcggaattcggaccgcgtgcgcttggcaaccgtagcattcttgcc

NGR234 nolO       GACCCTAGGCCAGCCACAAACAAGGATCGGATCAACGCGATTGTCAAGAAGCGCGAAGGC   1320
HH103 nolO HNU    GACCCTAGGCCGGCCACAAACAAGGATCGGATCAACGCGATTGTCAAGAAGCGCGAAGGC   1319
45436 nolO HNU    GACCCTAGGCCGGCCACAAACAAGGATCGGATCAACGCGATTGTCAAGAAGCGCGAAGGC   1319
25509 nolO HNU    GACCCTAGGCCGGCCACAAACAAGGATCGGATCAACGCGATTGTCAAGAAGCGCGAAGGC   1319
05684 nolO HNU    GACCCTAGGCCGGCCACAAACAAGGATCGGATCAACGCGATTGTCAAGAAGCGCGAAGGC   1319
05631 nolO HNU    GACCCTAGGCCGGCCACAAACAAGGATCGGATCAACGCGATTGTCAAGAAGCGCGAAGGC   1318
Consensus         gaccctaggcc gccacaaacaaggatcggatcaacgcgattgtcaagaagcgcgaaggc
```

```
NGR234 nolO      TATCGCCCCTTTGCGCCATCCGTGTTGGAGGAGGACGCCAACGAGTTCTTTGAATTGCCA  1380
HH103 nolO HNU   TATCGCCCCTTTGCGCCATCCGTGTTGGAGGAGGACGCCAACGAGTTCTTTGAATTGCCA  1379
45436 nolO HNU   TATCGCCCCTTTGCGCCATCCGTGTTGGAGGAGGACGCCAACGAGTTCTTTGAATTGCCA  1379
25509 nolO HNU   TATCGCCCCTTTGCGCCATCCGTGTTGGAGGAGGACGCCAACGAGTTCTTTGAATTGCCA  1379
05684 nolO HNU   TATCGCCCCTTTGCGCCATCCGTGTTGGAGGAGGACGCCAACGAGTTCTTTGAATTGCCA  1379
05631 nolO HNU   TATCGCCCCTTTGCGCCATCCGTGTTGGAGGAGGACGCCAACGAGTTCTTTGAATTGCCA  1378
Consensus        tatcgcccctttgcgccatccgtgttggaggaggacgccaacgagttctttgaattgcca

NGR234 nolO      GATAGCAGGCAGGAATTTCCCTTTATGAATTTCGTAGTGCCTGTGCGCGAATCGAAGCGC  1440
HH103 nolO HNU   GATAGCAGGCAGGAATTTCCCTTTATGAATTTCGTAGTGCCTGTGCGCGAATCCAAGCGC  1439
45436 nolO HNU   GATAGCAGGCAGGAATTTCCCTTTATGAATTTCGTAGTGCCTGTGCGCGAATCCAAGCGC  1439
25509 nolO HNU   GATAGCAGGCAGGAATTTCCCTTTATGAATTTCGTAGTGCCTGTGCGCGAATCCAAGCGC  1439
05684 nolO HNU   GATAGCAGGCAGGAATTTCCCTTTATGAATTTCGTAGTGCCTGTGCGCGAATCCAAGCGC  1439
05631 nolO HNU   GATAGCAGGCAGGAATTTCCCTTTATGAATTTCGTAGTGCCTGTGCGCGAATCCAAGCGC  1438
Consensus        gatagcaggcaggaatttccctttatgaatttcgtagtgcctgtgcgcgaatc aagcgc

NGR234 nolO      AATCTGCTCGGTGCCGTCACGCATGTCGATGGTACGGCGCGCTTGCAAACAGTATCGCGC  1500
HH103 nolO HNU   AATCTGCTCGGTGCCGTCACGCACGTCGATGGTACGGCGCGCTTGCAAACAGTATCGCGC  1499
45436 nolO HNU   AATCTGCTCGGTGCCGTCACGCACGTCGATGGTACGGCGCGCTTGCAAACAGTATCGCGC  1499
25509 nolO HNU   AATCTGCTCGGTGCCGTCACGCACGTCGATGGTACGGCGCGCTTGCAAACAGTATCGCGC  1499
05684 nolO HNU   AATCTGCTCGGTGCCGTCACGCACGTCGATGGTACGGCGCGCTTGCAAACAGTATCGCGC  1499
05631 nolO HNU   AATCTGCTCGGTGCCGTCACGCACGTCGATGGTACGGCGCGCTTGCAAACAGTATCGCGC  1498
Consensus        aatctgctcggtgccgtcacgca gtcgatggtacggcgcgcttgcaaacagtatcgcgc

NGR234 nolO      AACATCAATCAAGCGTATTGGGAGGTCATCAACGCCTTCAGGAAACGAACAGGTGTCCCG  1560
HH103 nolO HNU   AACATCAATCAAGCGTATTGGGAGGTCATCACCGCCTTTAGGAAACGAACAGGTGTCCCG  1559
45436 nolO HNU   AACATCAATCAAGCGTATTGGGAGGTCATCACCGCCTTTAGGAAACGAACAGGTGTCCCG  1559
25509 nolO HNU   AACATCAATCAAGCGTATTGGGAGGTCATCACCGCCTTTAGGAAACGAACAGGTGTCCCG  1559
05684 nolO HNU   AACATCAATCAAGCGTATTGGGAGGTCATCACCGCCTTTAGGAAACGAACAGGTGTCCCG  1559
05631 nolO HNU   AACATCAATCAAGCGTATTGGGAGGTCATCAACGCCTTCAGGAAACGAACAGGTGTCCCG  1558
Consensus        aacatcaatcaagcgtattgggaggtcatca cgcctt aggaaacgaacaggtgtcccg

NGR234 nolO      ATTCTGCTCAACACGTCCTTCAACAATAATGTTGAGCCGATAGTGGATTCGGTTGCGGAT  1620
HH103 nolO HNU   ATTCTGCTCAACACGTCCTTCAACAATAATGTTGAGCCGATAGTGGATTCGGTTGCGGAT  1619
45436 nolO HNU   ATTCTGCTCAACACGTCCTTCAACAATAATGTTGAGCCGATAGTGGATTCGGTTGCGGAT  1619
25509 nolO HNU   ATTCTGCTCAACACGTCCTTCAACAATAATGTTGAGCCGATAGTGGATTCGGTTGCGGAT  1619
05684 nolO HNU   ATTCTGCTCAACACGTCCTTCAACAATAATGTTGAGCCGATAGTGGATTCGGTTGCGGAT  1619
05631 nolO HNU   ATTCTGCTCAACACGTCCTTCAACAATAATGTTGAGCCGATAGTGGATTCGGTTGCGGAT  1618
Consensus        attctgctcaacacgtccttcaacaataatgttgagccgatagtggattcggttgcggat

NGR234 nolO      GCGGTGACCACATTTTTGACCACCGACTTGGATGGACTCGTGGTTGGGTCATATCTCATC  1680
HH103 nolO HNU   GCGGTGACCACATTTTTGACCACCGACTTGGATGGACTCGTGGTTGGGTCGTATCTCATC  1679
45436 nolO HNU   GCGGTGACCACATTTTTGACCACCGACTTGGATGGACTCGTGGTTGGGTCGTATCTCATC  1679
25509 nolO HNU   GCGGTGACCACATTTTTGACCACCGACTTGGATGGACTCGTGGTTGGGTCGTATCTCATC  1679
05684 nolO HNU   GCGGTGACCACATTTTTGACCACCGACTTGGATGGACTCGTGGTTGGGTCGTATCTCATC  1679
05631 nolO HNU   GCGGTGACCACATTTTTGACCACCGACTTGGATGGACTCGTGGTTGGGTCGTATCTCATC  1678
Consensus        gcggtgaccacatttttgaccaccgacttggatggactcgtggttgggtc tatctcatc

NGR234 nolO      AAGAAGCGGACTGCGTCACCGGAGGATTGGAGTAGACTTGCGCTTTCGTTGCCGCCTTAT  1740
HH103 nolO HNU   AAGAAGCGGACTGCGTCACCGGAGGACTGGAGTAGACTTGCGCTTTCGTTGCCGCCTTAT  1739
45436 nolO HNU   AAGAAGCGGACTGCGTCACCGGAGGACTGGAGTAGACTTGCGCTTTCGTTGCCGCCTTAT  1739
25509 nolO HNU   AAGAAGCGGACTGCGTCACCGGAGGACTGGAGTAGACTTGCGCTTTCGTTGCCGCCTTAT  1739
05684 nolO HNU   AAGAAGCGGACTGCGTCACCGGAGGACTGGAGTAGACTTGCGCTTTCGTTGCCGCCTTAT  1739
05631 nolO HNU   AAGAAGCGGACTGCGTCACCGGAGGACTGGAGTAGACTTGCGCTTTCGTTGCCGCCTTAT  1738
Consensus        aagaagcggactgcgtcaccggagga tggagtagacttgcgctttcgttgccgccttat

NGR234 nolO      TCAAGCCTCCATCAAGTGCGTGCATTTACGGCGTTAGATCGCCAAGAGACGGTATGCGAG  1800
HH103 nolO HNU   TCAAGCCTCCATCAAGTGCGTGCATTTACGGCGTTAGACCGCCAAGAGACGGTATGCGAG  1799
45436 nolO HNU   TCAAGCCTCCATCAAGTGCGTGCATTTACGGCGTTAGACCGCCAAGAGACGGTATGCGAG  1799
25509 nolO HNU   TCAAGCCTCCATCAAGTGCGTGCATTTACGGCGTTAGACCGCCAAGAGACGGTATGCGAG  1799
05684 nolO HNU   TCAAGCCTCCATCAAGTGCGTGCATTTACGGCGTTAGACCGCCAAGAGACGGTATGCGAG  1799
05631 nolO HNU   TCAAGCCTCCATCAAGTGCGTGCATTTACGGCGTTAGACCGCCAAGAGACGGTATGCGAG  1798
Consensus        tcaagcctccatcaagtgcgtgcatttacggcgttaga cgccaagagacggtatgcgag

NGR234 nolO      ATCCGCACGGTCCCTCCAGCCGAGAAGCCGTGCGGATTTCATCCGAGTTGTTCGAACTG  1860
HH103 nolO HNU   ATCCGCACGGTCCCTCCAGCCGAGAAGCCGTGCGGATTTCATCCGAGTTGTTCGAACTG  1859
45436 nolO HNU   ATCCGCACGGTCCCTCCAGCCGAGAAGCCGTGCGGATTTCATCCGAGTTGTTCGAACTG  1859
25509 nolO HNU   ATCCGCACGGTCCCTCCAGCCGAGAAGCCGTGCGGATTTCATCCGAGTTGTTCGAACTG  1859
05684 nolO HNU   ATCCGCACGGTCCCTCCAGCCGAGAAGCCGTGCGGATTTCATCCGAGTTGTTCGAACTG  1859
05631 nolO HNU   ATCCGCACGGTCCCTCCAGCCGAGAAGCCGTGCGGATTTCATCCGAGTTGTTCGAACTG  1858
Consensus        atccgcacggtccctccagccgagaagccgtgcggatttcatccgagttgttcgaactg

NGR234 nolO      CTAATGCGGATTGATGGAGAAGCTCCGCTCGGCGATATCTTGGACCTCATCGCGCCCAAT  1920
HH103 nolO HNU   CTAATGCGGATTGATGGAGAAGCTCCGCTCGGCGATATCTTGGACCTCATCGCGCCCAAT  1919
45436 nolO HNU   CTAATGCGGATTGATGGAGAAGCTCCGCTCGGCGATATCTTGGACCTCATCGCGCCCAAT  1919
25509 nolO HNU   CTAATGCGGATTGATGGAGAAGCTCCGCTCGGCGATATCTTGGACCTCATCGCGCCCAAT  1919
05684 nolO HNU   CTAATGCGGATTGATGGAGAAGCTCCGCTCGGCGATATCTTGGACCTCATCGCGCCCAAT  1919
05631 nolO HNU   CTAATGCGGATTGATGGAGAAGCTCCGCTCGGCGATATCTTGGACCTCATCGCGCCCAAT  1918
Consensus        ctaatgcggattgatggagaagctccgctcggcgatatcttggacctcatcgcgcccaat

NGR234 nolO      CAGAACCAGCCTGAAGCGCTCCTAAATGAACTGCGTGGGCTTTGGGAGCAGCGTAGCGTG  1980
HH103 nolO HNU   CAGAACCAGCCTGAAGCGCTCCTAAATGAACTGCGTGGGCTTTGGGAGCAGCGTAGCGTG  1979
45436 nolO HNU   CAGAACCAGCCTGAAGCGCTCCTAAATGAACTGCGTGGGCTTTGGGAGCAGCGTAGCGTG  1979
25509 nolO HNU   CAGAACCAGCCTGAAGCGCTCCTAAATGAACTGCGTGGGCTTTGGGAGCAGCGTAGCGTG  1979
05684 nolO HNU   CAGAACCAGCCTGAAGCGCTCCTAAATGAACTGCGTGGGCTTTGGGAGCAGCGTAGCGTG  1979
05631 nolO HNU   CAGAACCAGCTTGAAGCGCTCCTAAATGAACTGCGTGGGCTTTGGGAGCAGCGTAGCGTG  1978
Consensus        cagaaccagc tgaagcgctcctaaatgaactgcgtgggctttgggagcagcgtagcgtg
```

```
NGR234 nolO       CGACTGCATCCAATGCGCGCCGACTCTGCAGCGGAACCACTATCAAGTCCAATCAATCTG  2040
HH103 nolO HNU    CGACTGCATCCAATGCGCGCCGGCTCTGCAGCGGAACCACTATCAAGTTCAATCAATCTG  2039
45436 nolO HNU    CGACTGCATCCAATGCGCGCCGGCTCTGCAGCGGAACCACTATCAAGTTCAATCAATCTG  2039
25509 nolO HNU    CGACTGCATCCAATGCGCGCCGGCTCTGCAGCGGAACCACTATCAAGTTCAATCAATCTG  2039
05684 nolO HNU    CGACTGCATCCAATGCGCGCCGGCTCTGCAGCGGAACCACTATCAAGTTCAATCAATCTG  2039
05631 nolO HNU    CGACTGCATCCAATGCGCGCCGGCTCTGCAGCGGAACCACTATCAAGTTCAATCAATCTG  2038
Consensus         cgactgcatccaatgcgcgccg ctctgcagcggaaccactatcaagt caatcaatctg

NGR234 nolO       TA                                                            2042
HH103 nolO HNU    T.                                                            2040
45436 nolO HNU    T.                                                            2040
25509 nolO HNU    T.                                                            2040
05684 nolO HNU    T.                                                            2040
05631 nolO HNU    T.                                                            2039
Consensus         t
```

FIGURE S3. Alignment of homologous nucleotides sequence containing the putative *nolO* region of *Sinorhizobium fredii* strains (CCBAU45436, CCBAU25509, HH103), *S. sojae* CCBAU05684, *S.* sp. CCBAU05631, the *nolO* of *S.* sp. NGR234. The 1-bp deletions in *Sinorhizobium fredii* strains (CCBAU45436, CCBAU25509, HH103), *S. sojae* CCBAU05684, *S.* sp. CCBAU05631 sequences are indicated by black triangles.HNU means homologous nucleotides.

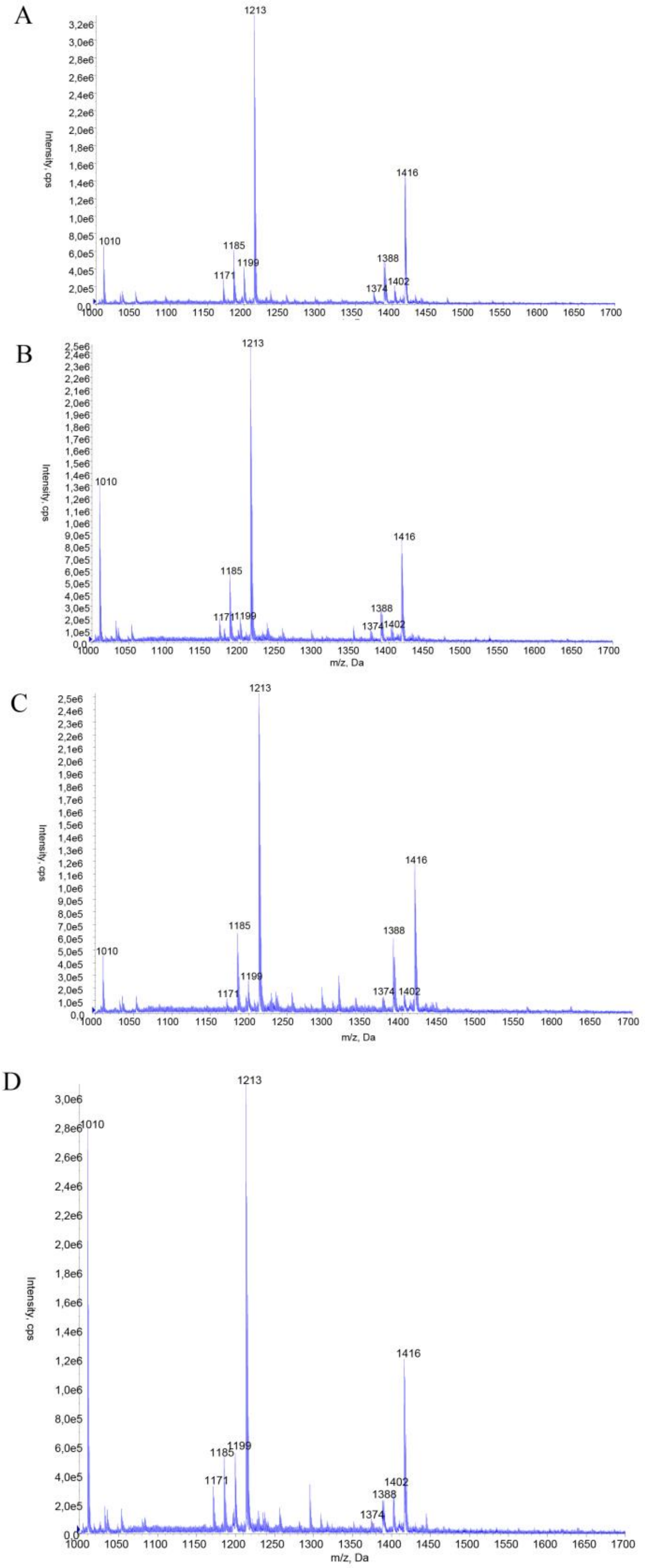

FIGURE S4. ESI-MS spectrum of LCOs secreted by four *Sinorhizobium* strains. The LCOs MS spectrums from *S. fredii* CCBAU 45436 **(A)**, *S. fredii* CCBAU25509 **(B)**, *S. sojae* CCBAU 05684 **(C)** and *Sinorhizobium* sp. CCBAU 05631**(D)** show the common compositions of Nod factors.

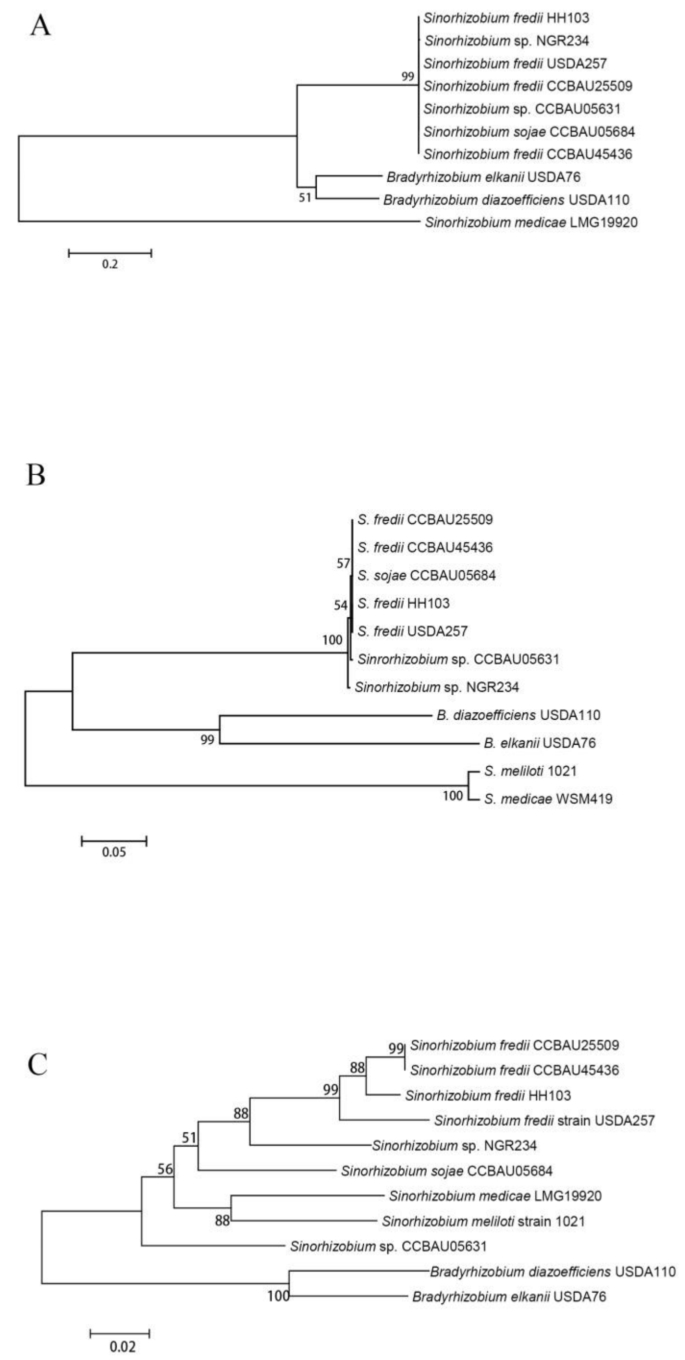

FIGURE S5. Phylogenetic tree of *noeI* (A), *nodA* (B), and *recA* (C) nucleotides of soybean nodulating microsymbionts and other *Sinorhizobial* strains. The tree was constructed using neighbor-joinng method. The evolutionary distances were computed using Maximum Composite Likelihood method. Scale bar indicateds 20% substitution of nucleotide **(A)**, 5% substitution of nucleotide **(B)** or 2% substitution of nucleotide **(C)**.

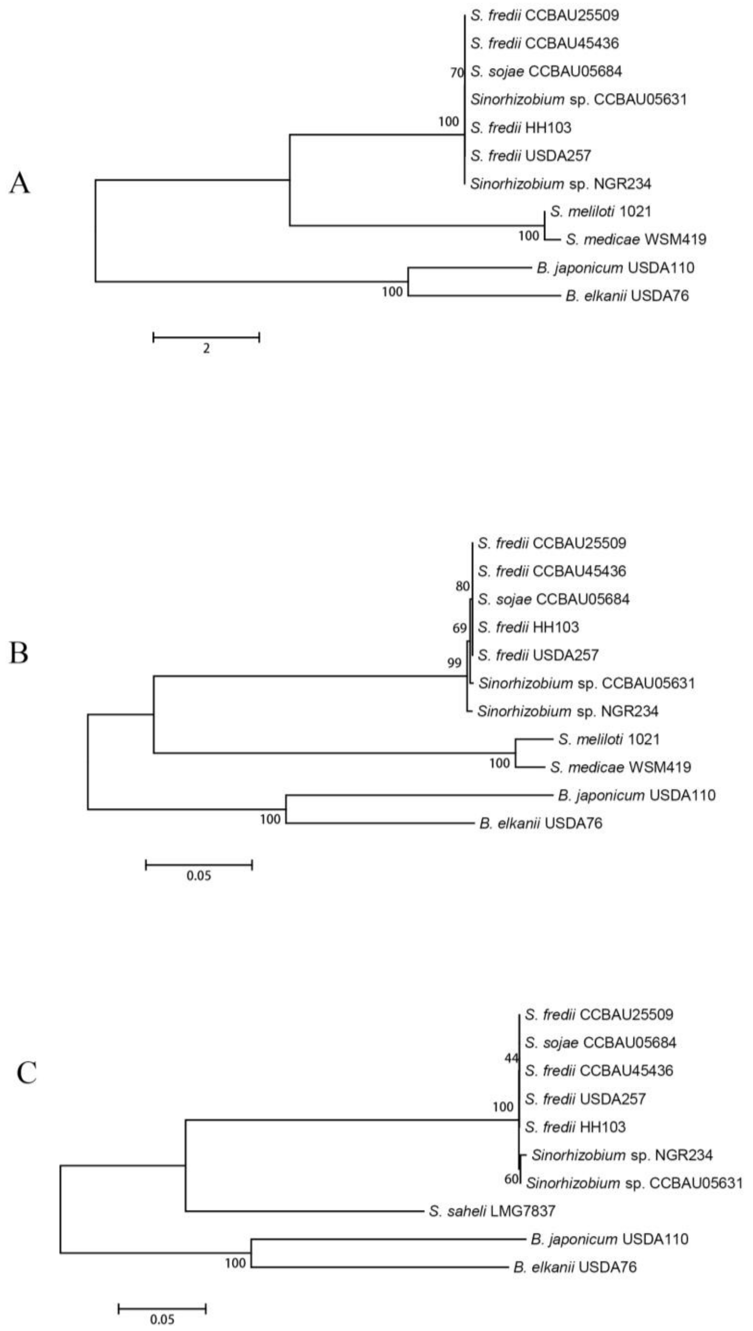

FIGURE S6. Phylogenetic tree of *nodB* (A), *nodC* (B), and *nodZ*(C) nucleotides of soybean nodulating microsymbionts and other *Sinorhizobial* strains. The tree was constructed using neighbor-joinng method. The evolutionary distances were computed using Maximum Composite Likelihood method. Scale bar indicateds 20% substitution of nucleotide **(A)**, 5% substitution of nucleotide (**B**), or 5% substitution of nucleotide (**C**).
